# Assessment of Strategies for Safe Drug Discontinuation and Transition of Denosumab Treatment in PMO—Insights From a Mechanistic PK/PD Model of Bone Turnover

**DOI:** 10.3389/fbioe.2022.886579

**Published:** 2022-06-17

**Authors:** Javier Martínez-Reina, José Luis Calvo-Gallego, Madge Martin, Peter Pivonka

**Affiliations:** ^1^ Departmento de Ingeniería Mecánica y Fabricación, Universidad de Sevilla, Seville, Spain; ^2^ CNRS, Univ Paris Est Creteil, Univ Gustave Eiffel, UMR 8208, MSME, Créteil, France; ^3^ School of Mechanical, Medical and Process Engineering, Queensland University of Technology, Brisbane, QLD, Australia

**Keywords:** postmenopausal osteoporosis, bone remodelling, bone mineralisation, denosumab discontinuation, RANK-RANKL-OPG pathway, PK-PD modelling, osteoclast precursors

## Abstract

Denosumab (Dmab) treatment against postmenopausal osteoporosis (PMO) has proven very efficient in increasing bone mineral density (BMD) and reducing the risk of bone fractures. However, concerns have been recently raised regarding safety when drug treatment is discontinued. Mechanistic pharmacokinetic-pharmacodynamic (PK-PD) models are the most sophisticated tools to develop patient specific drug treatments of PMO to restore bone mass. However, only a few PK-PD models have addressed the effect of Dmab drug holidays on changes in BMD. We showed that using a standard bone cell population model (BCPM) of bone remodelling it is not possible to account for the spike in osteoclast numbers observed after Dmab discontinuation. We show that inclusion of a variable osteoclast precursor pool in BCPMs is essential to predict the experimentally observed rapid rise in osteoclast numbers and the associated increases in bone resorption. This new model also showed that Dmab withdrawal leads to a rapid increase of damage in the bone matrix, which in turn decreases the local safety factor for fatigue failure. Our simulation results show that changes in BMD strongly depend on Dmab concentration in the central compartment. Consequently, bone weight (BW) might play an important factor in calculating effective Dmab doses. The currently clinically prescribed constant Dmab dose of 60 mg injected every 6 months is less effective in increasing BMD for patients with high BW (2.5% for 80 kg in contrast to 8% for 60 kg after 6 years of treatment). However, bone loss observed 24 months after Dmab withdrawal is less pronounced in patients with high BW (3.5% for 80kg and 8.5% for 60 kg). Finally, we studied how to safely discontinue Dmab treatment by exploring several transitional and combined drug treatment strategies. Our simulation results indicate that using transitional reduced Dmab doses are not effective in reducing rapid bone loss. However, we identify that use of a bisphosphonate (BP) is highly effective in avoiding rapid bone loss and increase in bone tissue damage compared to abrupt withdrawal of Dmab. Furthermore, the final values of BMD and damage were not sensitive to the time of administration of the BP.

## Introduction

Osteoporosis (OP) is a systemic skeletal disorder which is characterized by low bone mineral density (BMD) and a deteriorated bone microarchitecture which results in reduced bone strength, ultimately leading to an increase in fragility fractures (Lorentzon and Cummings, 2015). The age-dependent decrease in BMD is a strong indicator of the increase in fracture risk seen in the aging population (Kanis and Kanis, 1994; Felsenberg et al., 2002).

Currently, anti-resorptive agents are most widely used to treat osteoporosis (Anastasilakis et al., 2018). The most potent anti-resorptive agent is denosumab (Dmab) (Cummings et al., 2009), a monoclonal antibody to the receptor activator of nuclear factor-kB ligand (RANKL). RANKL is a key regulator of bone resorption by its effect on osteoclast development, function and survival. While Dmab has been shown to be effective in increasing BMD and reduce risk of fracture at major skeletal sites (Cummings et al., 2009; Papapoulos et al., 2015), a recent review on the effects of Dmab discontinuation (Anastasilakis et al., 2021) indicates that drug withdrawal leads to a rapid, significant increase in the concentrations of bone turnover markers (BTMs), in most cases to above pre-treatment baseline levels (Bone et al., 2011; Zanchetta et al., 2018).

It was also found that discontinuation of Dmab is typically associated with a decline in BMD throughout the skeleton. Indeed, the rate of BMD loss observed in patients who had stopped Dmab therapy (and did not receive any subsequent osteoporosis medication) was as high as 5–11% at all sites during the first year off-treatment (Miller et al., 2008; McClung et al., 2017; Popp et al., 2018; Zanchetta et al., 2018). Bone et al. showed that treatment of postmenopausal osteoporosis (PMO) with Dmab for 24 months subsequently followed for another 24 months off-treatment resulted in BMD loss at all skeletal sites that was evident at 6 months after the last injection (Bone et al., 2011). It was noted that BMD loss is bone site specific, with greatest BMD loss in the lumbar spine (LS) occurring at a mean of 18 months off-treatment, while both total hip (TH) and 1/3 radius (R) BMD continued to decline up to a mean of 30 months after the last injection (Bone et al., 2011).

It is well known that mechanistic pharmacokinetics-pharmacodynamics (PK-PD) models of OP treatments can be used to identify optimal treatment regimes. We and others have previously investigated the effect of Dmab dosing patterns on changes in BTMs, bone cell numbers and BMD (Marathe et al., 2011; Peterson and Riggs, 2012; Scheiner et al., 2014; Martínez-Reina and Pivonka, 2019). Based on these studies it was identified that inclusion of bone mineralisation is an essential model feature in order to predict BMD gains during Dmab treatment (Martínez-Reina and Pivonka, 2019). Recently, we also addressed the experimental finding of increased bone resorption after Dmab discontinuation using PK-PD modelling (Martínez-Reina et al., 2021a). Surprisingly, the bone cell population model (BCPM) of bone remodelling was not able to predict the rapid increase of active osteoclast numbers and the associated loss in BMD after Dmab drug holiday (Anastasilakis et al., 2021). Here we address the question on which model features need to be added to current state-of-the-art BCPMs in order to predict the effect of Dmab drug holiday on bone biomarkers. Given that in a clinical context it is essential to be able to switch drug treatments without detrimental effects to patients, such a model might be able to identify Dmab dosing regimens which minimise negative effects of drug holidays and/or change to a different drug treatment regimen.

As reviewed in Anastasilakis et al. (Anastasilakis et al., 2021), discontinuation of Dmab treatment is associated with a 3 to 5-fold higher risk for vertebral, major osteoporotic, and hip fractures (Lyu et al., 2020; Tripto-Shkolnik et al., 2020). This has been attributed to a given unopposed fracture risk similar to placebo-controlled trials. It was found that the off-treatment fracture risk among patients who had received Dmab was not different compared to the placebo group (Brown et al., 2013). However, amongst those discontinuing Dmab treatment a significant increase in multiple vertebral fractures were identified (Cummings et al., 2018). The fractures in this setting are typically clinical, occurring a few months after the effect of the last Dmab injection has been depleted (Anastasilakis et al., 2017) and are often described as rebound associated vertebral fractures (RAVFs). The review by Anastasilakis et al. summarizes RAVFs from these case reports starting from 2016 (see (Anastasilakis et al., 2021), Table 1).

In healthy subjects, a major purpose of bone remodelling is to remove old and damaged bone tissue (through the action of osteoclasts) and replace it with newly formed bone (laid down by osteoblasts) (Manolagas, 2018). This process is tightly regulated by osteocytes, i.e., terminally differentiated osteoblastic cells embedded in the bone matrix (Manolagas and Parfitt, 2013). Osteocytes regulate bone formation through expression of Wnt inhibitors including sclerostin (SOST) and Dickkopf-1 (DKK-1) (Schaffler et al., 2014; Tu et al., 2015), and bone resorption through expression of receptor activator of nuclear factor kappa-B ligand (RANKL) (Nakashima et al., 2011; Xiong et al., 2015) and its decoy receptor osteoprotegerin (OPG) (Udagawa et al., 2000). Other factors such as TGF-*β*, which is released from the bone matrix during osteoclastic bone resorption, regulate coupling between osteoclastic and osteoblastic cell populations (Matsuo and Irie, 2008).

Dmab binds RANKL with high affinity, and so prevents its binding to RANK receptors expressed on the surface of osteoclastic cells. Hence, Dmab suppresses osteoclast recruitment, maturation, function and survival, and significantly decreases bone resorption and associated bone loss (Kostenuik et al., 2009). As a bone anti-resorptive agent, its effect on osteoblasts is largely indirect through coupling of resorption and formation within the remodelling process.

In recent years, several investigators started with development of *in-silico* models of the action of Dmab in PMO with the aim to optimise and customise drug dosing. The majority of these models are based on mechanistic PK-PD models of drug treatments in osteoporosis (Lemaire et al., 2004; Pivonka et al., 2008; Scheiner et al., 2013; Martínez-Reina and Pivonka, 2019). A major finding of these models was that in order to be able to accurately model the effects of anti-resorptive drugs on BMD it is essential to take into account bone mineralisation as a physiological process (Scheiner et al., 2014; Martínez-Reina and Pivonka, 2019) together with accumulation of microcracks due to increased brittleness of the bone matrix (Martínez-Reina et al., 2021a; Martínez-Reina et al., 2021b).

As described above none of these BCPMs was able to reproduce the spike in bone resorption markers and associated rapid bone loss after Dmab discontinuation. Based on proposed molecular and cellular mechanisms for explaining this behaviour we analysed model structures of common BCPMs and identified that all current models are based on the assumption of a constant osteoclast precursor cell pool. This finding leads us to our major hypothesis for explaining effects of Dmab discontinuation:

• Overshoot in the active osteoclast population after removing remodelling suppression is linked to a variable osteoclast precursor pool;
• A secondary hypothesis is that differentiation of the uncommitted osteoclasts into the osteoclast precursors is regulated by RANKL.

In this paper, we extend our recent BCPM with respect to introducing osteoclast precursor cells as an additional cell pool of the osteoclastic linage. We first show that depending on the choice of RANKL regulation of the differentiation rates of uncommitted osteoclasts and osteoclast precursors this extended model can either represent the original model with a constant pool of precursors or represent new model features. We then test the hypothesis that introducing an additional cell pool for modelling the differentiation of osteoclastic cells is able to capture the following experimental observations after Dmab discontinuation: 1) BMD loss is dependent on the duration of Dmab treatment and 2) BMD loss is site specific, i.e. 18 months at the lumbar spine and 30 months at the hip.

This paper is organised as follows: In Section 2 we provide a detailed description of the mechanistic PK − PD model. The comparison of simulation results and experimentally observed changes in BMD are reported in Section 3, together with parametric studies of essential model parameters. The results are discussed in detail with respect to the clinical bone biology literature in Section 4. Conclusions and outlook to future work is presented in Section 5.

## Mechanistic PK-PD Model for Simulation of the Effect of Dmab on Bone Remodelling

### Model of Bone Cell Interactions in Bone Remodelling

**FIGURE 1:**
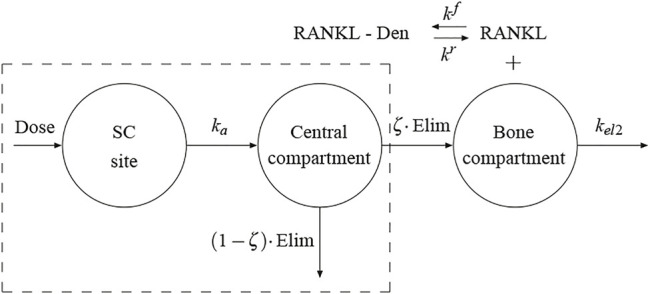
Schematic representation of the proposed PK model. The framed box represents the 1-compartment PK model proposed by Marathe et al. (Marathe et al., 2011). The bone compartment has been added here and the flux of Dmab from the central compartment to the bone compartment represents the production term in the competitive binding reactions between RANKL, OPG and Dmab.

Following the approach taken by Martin et al. (Martin et al., 2019), the bone remodelling process can be described as cell balance equations. The bone cell types (i.e. state variables) considered in the current model are: osteoblast precursor cells (Ob_p_), active osteoblasts (Ob_a_), osteoclast precursor cells (Oc_p_), active osteoclasts (Oc_a_) and osteocytes (Ot). The cell pools of uncommitted progenitor cells of both lineages (Ob_u_, Oc_u_) were assumed constant:

$$\frac{d\;{Ob}_{\text{p}}}{dt}=D_{{\text{Ob}}_{\text{u}}}\cdot \text{O}{\text{b}}_{\text{u}}\cdot \pi_{\text{a}\text{c}\text{t},\text{O}{\text{b}}_{\text{u}}}^{\text{TGF−β}}-D_{{\text{Ob}}_{\text{p}}}\cdot \text{O}{\text{b}}_{\text{p}}\cdot \pi_{\text{r}\text{e}\text{p},\text{O}{\text{b}}_{\text{p}}}^{\text{TGF−β}}$$ (1)

$$\begin{array}{l} +P_{{\text{Ob}}_{\text{p}}}\cdot \text{O}{\text{b}}_{\text{p}}\cdot \pi_{\text{a}\text{c}\text{t},\text{O}{\text{b}}_{\text{p}}}^{\text{Wnt}}\\ \frac{d\;{Ob}_{\text{a}}}{dt}=D_{{\text{Ob}}_{\text{p}}}\cdot \text{O}{\text{b}}_{\text{p}}\cdot \pi_{\text{r}\text{e}\text{p},\text{O}{\text{b}}_{\text{p}}}^{\text{TGF−β}}-\Delta_{{\text{Ob}}_{\text{a}}}\cdot {Ob}_{\text{a}} \end{array}$$ (2)

$$\frac{d\;{Oc}_{\text{p}}}{dt}=D_{{\text{Oc}}_{\text{u}}}\cdot \text{O}{\text{c}}_{\text{u}}\cdot \pi_{\text{a}\text{c}\text{t},\text{O}{\text{c}}_{\text{u}}}^{\mathrm{RANKL}}-D_{{\text{Oc}}_{\text{p}}}\cdot \text{O}{\text{c}}_{\text{p}}\cdot \pi_{\text{a}\text{c}\text{t},\text{O}{\text{c}}_{\text{p}}}^{\mathrm{RANKL}}$$ (3)

$$\frac{d\;{Oc}_{\text{a}}}{dt}=D_{{\text{Oc}}_{\text{p}}}\cdot \text{O}{\text{c}}_{\text{p}}\cdot \pi_{\text{a}\text{c}\text{t},\text{O}{\text{c}}_{\text{p}}}^{\mathrm{RANKL}}-A_{{\text{Oc}}_{\text{a}}}\cdot \text{O}{\text{c}}_{\text{a}}\cdot \pi_{\text{a}\text{c}\text{t},\text{O}{\text{c}}_{\text{p}}}^{\text{TGF−β}}$$ (4)

$$\frac{d\;Ot}{dt}=\eta \;\frac{d\;f_{bm}}{dt}$$ (5)

where $D_{{\text{Ob}}_{u}}$ , $D_{{\text{Ob}}_{p}}$ , $D_{{\text{Oc}}_{u}}$ and $D_{{\text{Oc}}_{p}}$ are the differentiation rates of Ob_u_, Ob_p_, Oc_u_ and Oc_p_, respectively; $A_{{\text{Oc}}_{a}}$ is the apoptosis rate of Oc_a_ and $\Delta_{{\text{Ob}}_{a}}$ is the rate of clearance of active osteoblasts through apoptosis or differentiation into osteocytes. The variables $\pi_{act,{Ob}_{u}}^{\mathrm{TGF-\beta}}$ , $\pi_{rep,{Ob}_{p}}^{\mathrm{TGF-\beta}}$ and $\pi_{act,{Oc}_{p}}^{\mathrm{TGF-\beta}}$ represent activator and repressor functions related to the binding of TGF-β to its receptor. Similarly, $\pi_{act,{Oc}_{u}}^{\mathrm{RANKL}}$ and $\pi_{act,{Oc}_{p}}^{\mathrm{RANKL}}$ are the activator functions related to the RANK-RANKL binding. Finally, $P_{{\text{Ob}}_{p}}$ is the proliferation rate of Ob_p_, a process which is mediated by the Wnt signalling pathway through the activator function $\pi_{act,Ob_{p}}^{\mathrm{Wnt}}$ and is described in detail in the Supplementary Material along with other features of the model. A schematic figure of the mechanistic PK-PD model is presented in Figure 1. Model parameters of the cell population model are given in the Supplementary Table S1.

Equation 5 establishes that the population of osteocytes varies proportional to the bone matrix fraction *f*_*bm*_, given that the density of osteocytes is constant within the bone matrix, *η*, is assumed constant, as done in (Martin et al., 2019). Finally, the variation of bone matrix fraction is obtained through the balance between resorbed and formed tissue:

$$\frac{df_{bm}}{dt}=-k_{\mathrm{res}}\cdot {Oc}_{\text{a}}+k_{\mathrm{form}}\cdot {Ob}_{\text{a}}$$ (6)

where *k*_*res*_ and *k*_*form*_ are, respectively, the rates of bone resorption and osteoid formation (see Supplementary Table S1).

### Competitive RANK-RANKL-OPG Binding. Action of Dmab

The RANK-RANKL-OPG signalling pathway controls the differentiation of uncommitted osteoclast progenitors and osteoclasts maturation, respectively through $\pi_{act,Oc_{u}}^{\mathrm{RANKL}}$ and $\pi_{act,Oc_{p}}^{\mathrm{RANKL}}$ (see Eqs. 3, 4. Thus, an imbalance in that pathway, such as that occurring after menopause, may result in the development of osteoporosis. The action of Dmab against the disease aims at restoring that balance. Following Martin et al. (Martin et al., 2019), the concentrations of OPG, RANK and RANKL are given by the following equations, which are explained in the Supplementary Material:

$$\left[OPG\right]=\frac{P_{\text{OPG}}}{{\tilde{D}}_{\text{OPG}}+\frac{{\tilde{D}}_{\text{OPG}-\text{RANKL}}\;\left[RANKL\right]}{K_{\text{OPG}-\text{RANKL}}}}$$ (7)

$$\left[RANK\right]=\frac{N_{{\text{Oc}}_{\text{p}}}^{\text{RANK}}\;{Oc}_{\text{p}}}{1+\frac{\left[RANKL\right]}{K_{\text{RANK}-\text{RANKL}}}}$$ (8)

$$\begin{array}{ll} \left[RANKL\right]= & P_{\text{RANKL}}\cdot \left[{\tilde{D}}_{\text{RANKL}}+\frac{{\tilde{D}}_{\text{OPG}-\text{RANKL}}}{K_{\text{OPG}-\text{RANKL}}}\cdot \left[OPG\right]\right.\\ & {\left.+\frac{{\tilde{D}}_{\text{RANK}-\text{RANKL}}}{K_{\text{RANK}-\text{RANKL}}}\cdot \left[RANK\right]+\frac{{\tilde{D}}_{\text{Dmab}-\text{RANKL}}}{K_{\text{RANKL}-\text{Dmab}}}\cdot {\left[D\text{m}\text{a}\text{b}\right]}_{\text{BC}}\right]}^{-1} \end{array}$$ (9)

where the binding of Dmab to RANKL is considered in the last term of the bracket of Eq. 9; ${\tilde{D}}_{\text{X}}$ and ${\tilde{D}}_{\text{X}-\text{Y}}$ are the degradation rates of the factor X and the complex X-Y, respectively; *K*_X-Y_ is the dissociation constant of the complex X-Y and $N_{{\text{Oc}}_{p}}^{\text{RANK}}$ is the number of RANK receptors per osteoclast precursor. [*Dmab*]_BC_ is the concentration of Dmab in the bone compartment and will be analysed in Section 2.3. *P*_OPG_ is the production rate of OPG by active osteoblasts:

$$P_{\text{OPG}}=\beta_{{\text{OPG,Ob}}_{\text{a}}}\;\pi_{{\text{rep,Ob}}_{\text{a}}}^{\text{PTH}}\;{Ob}_{\text{a}}\;\left(1-\frac{\left[OPG\right]}{\left[{OPG}_{\max}\right]}\right)$$ (10)

where $\beta_{{\text{OPG,Ob}}_{a}}$ is the OPG production rate, $\pi_{{\text{rep,Ob}}_{a}}^{\text{PTH}}$ is the repressor function that quantifies the effect of PTH on the production of OPG and [OPG_max_] is the saturation concentration of OPG above which no further production takes place. To evaluate *P*_RANKL_, the RANKL production rate of Eq. 9, we have assumed that RANKL is expressed by osteocytes and osteoblasts precursors, following experimental evidence (Nakashima et al., 2011; Xiong et al., 2015) and then:

$$\begin{array}{ll} P_{\text{RANKL}} & =\beta_{\text{RANKL,Ot}}\;Ot\;\left(1-\frac{{\left[RANKL\right]}_{\mathrm{tot}}}{{\left[RANKL\right]}_{\max}}\right)\;\pi_{\text{act,RANKL}}^{\text{dam}}\\ & +\beta_{{\text{RANKL,Ob}}_{\text{p}}}\;\pi_{\text{act/rep,RANKL}}^{\text{PTH,NO}}\;{Ob}_{\text{p}}\;\left(1-\frac{{\left[RANKL\right]}_{\mathrm{tot}}}{{\left[RANKL\right]}_{\max}}\right)+P_{\text{RANKL}}^{\text{PMO}} \end{array}$$ (11)

where $P_{\text{RANKL}}^{\text{PMO}}$ is the RANKL production due to PMO; *β*_RANKL,Ot_ and $\beta_{{\text{RANKL,Ob}}_{p}}$ are the RANKL production rate of osteocytes and osteoblast precursors, respectively; $\pi_{\text{act/rep,RANKL}}^{\text{PTH,NO}}$ is a co-regulatory function that takes into account the up-regulation of RANKL transcription by the parathyroid hormone (PTH) and its inhibition by nitric oxide (NO) (Martin et al., 2019) and $\pi_{\text{act,RANKL}}^{\text{dam}}$ is an activator function accounting for the upregulation of RANKL expression by osteocytes due to microstructural damage (Martínez-Reina et al., 2021b) (see the details of both regulatory functions in the Supplementary Material). Finally, [RANKL]_max_ is the saturation concentration of RANKL above which no further expression takes place and [RANKL]_*tot*_ is the total concentration of RANKL (bound and free) and is defined as follows:

$$\begin{array}{ll} {\left[RANKL\right]}_{\mathrm{tot}} & =\left[RANKL\right]\cdot \\ & \left(1+\frac{\left[OPG\right]}{K_{\text{OPG}-\text{RANKL}}}+\frac{\left[RANK\right]}{K_{\text{RANK}-\text{RANKL}}}+\frac{{\left[Dmab\right]}_{\text{BC}}}{K_{\text{RANKL}-\text{Dmab}}}\right) \end{array}$$ (12)

Following (Martínez-Reina et al., 2021b), the RANKL production due to PMO, $P_{\text{RANKL}}^{\text{PMO}}$ is modelled as a sigmoidal function of time:

$$P_{\text{RANKL}}^{\text{PMO}}\left(t\right)=\left\{\begin{array}{cc} P_{\text{RANKL},\max}^{\text{PMO}}\;\frac{{\left(t-t_{\text{onset}}\right)}^{\gamma }}{{\left(t-t_{\text{onset}}\right)}^{\gamma }+\delta_{\text{PMO}}^{\gamma }}\; & \;for\;t\geq t_{\text{onset}}\\ 0\; & \;for\;t<t_{\text{onset}} \end{array}\right.$$ (13)

where $P_{\text{RANKL},\max}^{\text{PMO}}$ is the maximum (long-term) RANKL production due to PMO, *t*_onset_ is the time of onset of the disease, *δ*_PMO_ is a time constant establishing when the 50% of $P_{\text{RANKL},\max}^{\text{PMO}}$ is reached and *γ* is the sigmoidicity factor.

Equations 11–13 can be substituted in Eq. 9 to work out the free RANKL concentration, i.e. [RANKL]. Then, the activator functions in Eqs. 3, 4 have the same structure:

$$\pi_{\mathrm{act,X}}^{\text{RANKL}}=\frac{\left[RANKL\right]}{K_{\mathrm{act,X}}^{\mathrm{RANKL}}+\left[\mathrm{RANKL}\right]}\;\text{with X }=\;{Oc}_{\text{u}},\;\text{O}{\text{c}}_{\text{p}}$$ (14)

and different constants: $K_{act,Oc_{u}}^{\text{RANKL}}$ and $K_{act,Oc_{p}}^{\text{RANKL}}$ . In previous works (Martínez-Reina and Pivonka, 2019; Martínez-Reina et al., 2021a; Martínez-Reina et al., 2021b) both constants $K_{\mathrm{act,X}}^{\text{RANKL}}$ were chosen equal in Eq. 3, so resulting in a constant pool of Oc_p_ in the steady state. One of the novelties of the current paper is to choose these constants different, so creating a variable pool of Oc_p_. To analyse the effect of this change, the response of two models is compared: 1) the model proposed in this paper, termed as *BCPM*_*ext*_ (extended model) with different constants $K_{\mathrm{act,X}}^{\text{RANKL}}$ for Oc_*u*_ and Oc_*p*_, leading to a variable pool of Oc_p_ and 2) a model, termed as *BCPM*_*st*_ (standard model) that coincides with the new model except that constants *K*_act,X_ are equal and thus produces a constant pool of Oc_p_.

### Two-Compartment PK Model of Dmab

Several pharmacokinetic (PK) models of Dmab have been proposed including one- and two-compartment models (Dua et al., 2015). In recent papers (Martínez-Reina and Pivonka, 2019; Martínez-Reina et al., 2021a) we have used a one-compartment model with Michaelis-Menten kinetics in order to characterise the serum Dmab PK profiles, with the constants of that model fitted from a clinical study by Marathe et al. (Marathe et al., 2011). This model is framed in a dashed box in Figure 1. A first-order rate process (*k*_*a*_) governs the absorption of the drug (*Dose*) from the subcutaneous (SC) injection site into the central compartment ([*Dmab*]_*CC*_), being *V*_*c*_/*F* the volume of the central compartment adjusted for bioavailability. The drug elimination from the central compartment is described by a combination of a linear first-order process (*k*_*el1*_) and a non-linear saturation process (*V*_max_, *K*_*m*_):

$$\frac{d{\left[Dmab\right]}_{\text{C}\text{C}}}{dt}=\frac{Dose}{V_{c}/F}\;k_{a}\;e^{-k_{a}\;t}-\left[k_{\mathrm{el1}}\;{\left[Dmab\right]}_{\text{C}\text{C}}+\frac{V_{max}}{V_{c}/F}\;\frac{{\left[Dmab\right]}_{\text{C}\text{C}}}{K_{m}+{\left[Dmab\right]}_{\text{C}\text{C}}}\right]$$ (15)

In Eq. 15 *Dose* is given in ng per kg of body weight and then [Dmab]_*CC*_ is calculated in ng/ml and subsequently converted into pmol/l, through the molecular weight of Dmab *M*_*Dmab*_ = 149 kDa (Amgen). Supplementary Table S1 summarises the PK model parameters of the one-compartment model adjusted by Marathe et al. (Marathe et al., 2011).

In previous models (Martínez-Reina and Pivonka, 2019; Martínez-Reina et al., 2021a) we have assumed that a fraction of the Dmab present in the central compartment was available in the bone compartment to compete with RANK to bind to RANKL. However, this availability actually implied a reversible exchange of Dmab between both compartments. Given the affinity of Dmab for RANKL, which is expressed by osteoblasts precursors within the bone compartment, a flux from the central compartment to the bone compartment seems more plausible than a reversible exchange. To this end, we have added the bone compartment to the PK model (see Figure 1). The term in square brackets in Eq. 15 represents the elimination from the central compartment in the model of Marathe et al. We have assumed that only a fraction (1 − *ζ*) of this term is actually eliminated via urine and the rest, *ζ*, is the flux of Dmab into the bone compartment. In turn, the latter fraction can be considered as $P_{Dmab_{BC}}$ , the production term of Dmab in the competitive binding reactions between RANKL, OPG and Dmab:

$$P_{{\text{Dmab}}_{\text{B}\text{C}}}=\zeta \;\left[k_{\mathrm{el1}}\;{\left[Dmab\right]}_{\text{C}\text{C}}+\frac{V_{max}}{V_{c}/F}\;\frac{{\left[Dmab\right]}_{\text{C}\text{C}}}{K_{m}+{\left[Dmab\right]}_{\text{C}\text{C}}}\right]$$ (16)

This production rate can be replaced in the expression that gives the concentration of ligands in competitive binding reactions (see Supplementary Material), i.e.:

$${\left[Dmab\right]}_{\text{B}\text{C}}=\frac{P_{{\text{Dmab}}_{\text{B}\text{C}}}}{{\tilde{D}}_{{\text{Dmab}}_{\text{B}\text{C}}}+\frac{{\tilde{D}}_{\text{RANKL}-\text{Dmab}}}{K_{\text{RANKL}-\text{Dmab}}}\cdot \left[RANKL\right]}$$ (17)

This expression can be used in Eq. 9 to give the concentration of free RANKL. All the parameters are known from previous works (Martínez-Reina and Pivonka, 2019), except for ${\tilde{D}}_{{\text{Dmab}}_{BC}}$ and *ζ* that needs to be adjusted in this model as explained later on.

### Damage

Targeted bone remodelling theories hypothesise that one of the major functions of bone remodelling is to remove microcracks from bone matrix, so avoiding an excessive accumulation of the latter, which could result in macroscopic failure (Parfitt, 2002). The accumulation of microcracks in a particular volume of material is addressed here using a Continuum Damage Mechanics approach (Lemaitre and Chaboche, 1990). This theory introduces a damage variable, *d*, which is linked to the density of microcracks in a volume of material and to the loss of stiffness through Eq. 18. This variable is such that *d* ∈ [0, 1], with *d* = 0 corresponding to an undamaged state and *d* = 1 to a local fracture or failure situation:

$$C=\left(1-d\right)\;C_{0}$$ (18)

where **C** and **C**_0_ are, respectively, the stiffness tensors of damaged and undamaged bone (Lemaitre and Chaboche, 1990). In the isotropic damage theory, Eq. 18 can be rewritten in terms of the respective Young’s moduli, *E* and *E*_0_, as *E* = (1 − *d*) *E*_0_ (Pattin et al., 1996; Zioupos and Currey, 1998) (see Eq. 38).

A balance of microdamage is considered through the accumulation due to fatigue loading and the removal due to bone remodelling, as osteoclasts resorb the damaged tissue, while the osteoid deposited by osteoblasts is initially intact. The evolution law for damage can be expressed as:

$$\dot{d}={\dot{d}}_{A}-{\dot{d}}_{R}$$ (19)

where ${\dot{d}}_{A}$ is the rate of damage accumulation by fatigue loading and ${\dot{d}}_{R}$ is the rate of damage removal by bone remodelling. The latter is assessed by assuming that damage is uniformly distributed throughout the representative volume element (RVE). So, the amount of repaired damage is proportional to the damage present in that volume and to the volume of tissue being resorbed, ${\dot{V}}_{r}$ , through the fraction that this volume represents within the bone matrix volume:

$${\dot{d}}_{R}=d\;\frac{{\dot{V}}_{r}}{V_{bm}}=d\;\frac{k_{\mathrm{res}}\cdot Oc_{a}}{f_{bm}}$$ (20)

Damage accumulation is evaluated following the procedure described in (Martínez-Reina et al., 2008; Martínez-Reina et al., 2009). This procedure makes use of the results of the experimental fatigue tests performed by Pattin et al. (Pattin et al., 1996), who provided the evolution of damage with the strain level and the number of cycles. This evolution was mathematically modelled by García-Aznar et al. (García-Aznar et al., 2005) to yield the following differential equation under tensile stresses:

$${\dot{d}}_{a}=\dot{N}\;\frac{C_{1}}{C_{2}\;\gamma_{f}}\;{\left(1-d\right)}^{1-\gamma_{f}}\;\varepsilon_{max}^{\delta_{f}}\;\exp\left(-C_{2}\;{\left(1-d\right)}^{\gamma_{f}}\right)$$ (21)

where $\dot{N}$ is the number of cycles applied per unit time and *ɛ*_max_ is the maximum principal strain expressed in *μɛ*.^1^ The rest of parameters and constants of the model are:

$$\begin{array}{cc} C_{1}=\frac{e^{C_{2}}-1}{K_{f}\left(\left[Ca\right]\right)}; & \;\delta_{f}=14.1;\\ \gamma_{f}=-0.018\left(\varepsilon_{max}-4100\right)+12; & \;C_{2}=-20; \end{array}$$ (22)

where *K*_*f*_([*Ca*]) is a function of the mineral content which will be defined next. The experimental tests perfomed by Pattin et al. (Pattin et al., 1996) included an estimation of fatigue life, *N*_*f*_, which was related to the deformation by the following expression:

$$N_{f}=\frac{K_{f}}{\varepsilon_{max}^{\delta_{f}}}$$ (23)

where *K*_*f*_ was assumed constant and equal to 1.445 ⋅ 10^53^ in tension. Martínez-Reina et al. (Martínez-Reina et al., 2008) introduced a correction in *K*_*f*_ to consider the degradation of the fatigue properties with the increase in mineral content. A life *N*_*f*_ = 10^7^ cycles was assigned to the fatigue limit, which is usually assumed to occur for a given fraction of the ultimate tensile strain, *ɛ*_*u*_/*β*, where the parameter *β* depends on the type of material (Juvinall, 1967) and *β* = 2 was assumed for bone (Martínez-Reina et al., 2008) with good results. So, *K*_*f*_ was obtained from Eq. 23 as:

$$K_{f}\left(\left[Ca\right]\right)=10^{7}\;{\left(\frac{\varepsilon_{u}\left(\left[Ca\right]\right)}{\beta }\right)}^{\delta_{f}}$$ (24)

where the ultimate tensile strain depends on the calcium concentration of bone matrix, [*Ca*], as Currey (Currey, 2004) showed. The following regression was fitted in (Martínez-Reina et al., 2008) from the experimental results presented by Currey (Currey, 2004):

$$log\;\varepsilon_{u}=31.452-11.341\;log\;\left[Ca\right]$$ (25)

where *ɛ*_*u*_ is expressed in *μɛ* and the concentration [*Ca*] is expressed in mg of calcium per g of bone matrix. This concentration is directly related to the ash fraction, *α*, which will be defined in the next section. More precisely, the relation [*Ca*] = 398.8 *α* was assumed, based on the molecular weigths of hydroxyapatite and type I collagen (Martínez-Reina et al., 2008).

### Algorithm of Bone Mineralisation

The mineralisation model used in this work is based on that presented in (Martínez-Reina et al., 2008) and implemented in a model which is similar to the present one (Martínez-Reina and Pivonka, 2019). That model was a mixture of differential and recursive equations, difficult to implement in a system of ODEs. For this reason, it has been simplified to yield an explicit set of differential equations, explained next.

Bone is made up of a solid bone matrix and pores filled with marrow. A certain representative volume element, *V*_*RVE*_, can be divided into the bone matrix volume, *V*_*bm*_, and the volume of pores, *V*_*p*_. The bone matrix volume is divided into inorganic (mineral), organic (mainly collagen) and water phases, designated as *V*_*m*_, *V*_*o*_ and *V*_*w*_, respectively:

$$V_{\mathrm{RV E}}=V_{bm}+V_{p}=V_{m}+V_{o}+V_{w}+V_{p}$$ (26)

The composition of bone matrix is defined in terms of the volume fractions of the three phases as:

$$v_{i}=\frac{V_{i}}{V_{bm}}\;\forall i=m,o,w$$ (27)

Thus, the following condition holds

$$v_{m}+v_{o}+v_{w}=1$$ (28)

The mineral content is usually measured by the so-called ash fraction, the ratio between mass of mineral (or ash mass) and dry mass (the sum of inorganic and organic mass):

$$\alpha =\frac{m_{m}}{m_{m}+m_{o}}=\frac{\rho_{m}\;V_{m}}{\rho_{m}\;V_{m}+\rho_{o}\;V_{o}}=\frac{\rho_{m}\;v_{m}}{\rho_{m}\;v_{m}+\rho_{o}\;v_{o}}$$ (29)

where Eq. 27 have been used and *ρ*_*i*_ are the corresponding densities of the three phases, being *ρ*_*m*_ = 3.2 g/cm^3^ (Currey, 2004) and *ρ*_*o*_ = 1.41 g/cm^3^ (Hernandez et al., 2001a). The tissue density is then given by:

$$\rho_{t}=\frac{m_{w}+m_{m}+m_{o}}{V_{bm}}=\rho_{w}\;v_{w}+\rho_{m}\;v_{m}+\rho_{o}\;v_{o}=1+\left(\rho_{o}-1\right)\;v_{o}+\left(\rho_{m}-1\right)\;v_{m}$$ (30)

where *ρ*_*w*_ = 1 g/cm^3^ and Eqs. 27, 28 have been used to derive the right-hand side of Eq. 30.

Osteoid, the tissue laid by osteoblasts, contains only the organic phase and no mineral. Mineral accumulates in bone matrix afterwards, during the mineralisation process, which consists of three phases: 1) an initial phase, called mineralisation lag time, that lasts from 6 to 22 days (Eriksen et al., 1990; Need et al., 2007) during which no deposition of mineral occurs; 2) a primary phase, which is very quick (it takes a few days to reach the 70% of the maximum mineral content (Hernandez et al., 2001b)), and 3) a secondary phase, when mineral is added at a decreasing rate (Parfitt, 1983), as the tissue becomes saturated with mineral. Mineral accumulates in bone matrix by displacing water (Hernandez et al., 2001a). Thus, the volume fraction of organic phase is approximately constant during the mineralisation process and fixed here at *v*_*o*_ = 3/7 (Martin, 1984); while the variations of mineral and water volume fractions would hold Δ*v*_*m*_ = −Δ*v*_*w*_. So, the mineralisation process is accounted for through the temporal variation of *v*_*m*_. As $v_{m}=\frac{V_{m}}{V_{bm}}$ (recall Eq. 27), the temporal derivative of this expression gives:

$${\dot{v}}_{m}=\frac{{\dot{V}}_{m}}{V_{bm}}-v_{m}\;\frac{{\dot{V}}_{bm}}{V_{bm}}={\left.{\dot{v}}_{m}\right)}_{V_{bm}=constant}-v_{m}\;\frac{{\dot{V}}_{bm}}{V_{bm}}$$ (31)

The first term corresponds to the variation of the mineral content due to mineralisation or resorption and it would be equal to the variation of mineral content if *V*_*bm*_ were constant. The second term is due to the variation of porosity. To understand this term it must be noted that the mineral content decreases when osteoid is deposited, since osteoid does not contain any mineral and it only contributes to increase the bone matrix volume, so reducing the concentration of mineral. Both terms will be analysed separately:

$${\left.{\dot{v}}_{m}\right)}_{V_{bm}=cte}={\left.{\dot{v}}_{m}\right)}_{mineralisation}-{\left.{\dot{v}}_{m}\right)}_{resorption}$$ (32)

In a previous model (Martínez-Reina et al., 2008; Martínez-Reina and Pivonka, 2019) the variation of *v*_*m*_ due to mineralisation was defined in a piecewise manner, considering the three phases separately: with no variation of *v*_*m*_ during the mineralisation lag time, a linear increase during the primary phase and an exponential increase during the secondary phase. Here, this procedure will be simplified by assuming that it is governed by a saturation model:

$${\left.{\dot{v}}_{m}\right)}_{mineralisation}=K\;\left(v_{m}^{max}-v_{m}\right)$$ (33)

leading to an exponential solution which approximates rather well the global response of the previous model for a value of the constant *K* = 0.007, with a fast initial mineralisation rate (primary phase) that slows down in the mid-long-term (secondary phase). The maximum mineral content $v_{m}^{\max}=0.516$ was fixed such that, together with the aforementioned *v*_*o*_ = 3/7, it yields the maximum tissue density $\rho_{t}^{\max}=2.31\;{g/cm}^{3}$ (Hernandez et al., 2001a) through Eq. 30. The rate constant *K* will be assumed fixed in this work, though it may depend on the amount of calcium and phosphorus available in the serum, which, in turn, may depend on diverse physiological factors (Peterson and Riggs, 2010).

The variation of mineral content due to resorption is similar to the damage repair term in the damage model (see Eq. 20). It must be taken into account that mineral is dissolved by osteoclasts and so removed from the bone matrix as damage was previously assumed. Thus, the amount of mineral removed by resorption is proportional to the mineral content of the tissue being resorbed, *v*_*m*_, and to the proportion of tissue being resorbed within the bone matrix, $\frac{{\dot{V}}_{r}}{V_{bm}}$ :

$${\left.{\dot{v}}_{m}\right)}_{resorption}=v_{m}\;\frac{{\dot{V}}_{r}}{V_{bm}}=v_{m}\;\frac{{\dot{V}}_{r}/V_{\mathrm{RV E}}}{V_{bm}/V_{\mathrm{RV E}}}=v_{m}\;\frac{{\dot{V}}_{r}/V_{\mathrm{RV E}}}{f_{bm}}=\frac{v_{m}}{f_{bm}}\;k_{\mathrm{res}}\;Oc_{a}$$ (34)

Using Eqs. 32-34 Eq. 31 can be rewritten as:

$${\dot{v}}_{m}=K\;\left(v_{m}^{max}-v_{m}\right)-\frac{v_{m}}{f_{bm}}\;k_{\mathrm{res}}\;Oc_{a}-v_{m}\;\frac{{\dot{f}}_{bm}}{f_{bm}}$$ (35)

where it has been used that $\frac{{\dot{V}}_{bm}}{V_{bm}}=\frac{{\dot{f}}_{bm}}{f_{bm}}$ . Taking into account the balance between formation and resorption (Eq. 6):

$${\dot{v}}_{m}=K\;\left(v_{m}^{max}-v_{m}\right)-\frac{v_{m}}{f_{bm}}\;k_{\mathrm{form}}\;Ob_{a}$$ (36)

Once *v*_*m*_ is updated, the ash fraction can be derived from Eq. 29 and the tissue density from Eq. 30. Then, the apparent density is given by:

$$\rho =\frac{m_{w}+m_{m}+m_{o}}{V_{\mathrm{RV E}}}=\frac{m_{w}+m_{m}+m_{o}}{V_{bm}}\;\frac{V_{bm}}{V_{\mathrm{RV E}}}=\rho_{t}\;f_{bm}$$ (37)

Bone was assumed to be an isotropic material with a Poisson’s ratio *ν* = 0.3 and a Young’s modulus given in MPa by the following expresions:

$$E\left(\rho ,d\right)=\left\{\begin{array}{cc} 2014\;\rho^{2.5}\;\left(1-d\right)\; & \;if\;\;\rho <1.2\;g/cm^{3}\\ 1763\;\rho^{3.2}\;\left(1-d\right)\; & \;if\;\;\rho \geq 1.2\;g/cm^{3} \end{array}\right.$$ (38)

These are based on the correlations experimentally obtained by Jacobs (Jacobs, 1994), which were multiplied by the factor (1 − *d*) to consider microstructural damage as usually done in Continuum Damage Mechanics (Lemaitre and Chaboche, 1990).

### Variables Used to Measure the Effect of the Treatments

The main effect of the treatment is the bone density gain (BDG), which is measured with respect to baseline, i.e. at the beginning of treatment, when bone apparent density is *ρ*_0_:

$$BDG\left(\%\right)=\frac{\rho \left(t\right)-\rho_{0}}{\rho_{0}}\cdot 100$$ (39)

The monthly time derivative of apparent density (*MTDAD*) is defined in Eq. 40 and used to measure the intensity of bone loss and, more precisely, the spike after Dmab discontinuation, as bone loss is accelerated a few months after the last Dmab injection, as compared to the disease state before treatment:

$$MTDAD\left(\%\;{month}^{-1}\right)=\frac{\dot{\rho }}{\rho \left(t=0\right)}\cdot 100$$ (40)

Finally, the fracture risk is evaluated by assessing the critical load *σ* ^*crit*^(*t*), i.e. the overload that would lead to local fracture (*d* = 1) in a few days for a given bone. This overload would be given by the present model as a function of the porosity, mineral content, bone turnover rate and current damage level. So, for a given instant *t*, the overload is calculated by integrating Eq. 19 and using the following algorithm:

1. Extract the results *f*_*bm*_(*t*), *d*(*t*), *Oc*_*a*_(*t*) and *α*(*t*) for the considered instant *t*.
2. Assume *f*_*bm*_(*t*), *Oc*_*a*_(*t*) and *α*(*t*) as constants and consider *d*(*t*) = *d*_0_ as the initial condition for the integration of Eq. 19. This equation is integrated over time *τ* > *t*.
3. Evaluate the apparent density, *ρ*(*t*), from *f*_*bm*_(*t*) and *α*(*t*) by using Eqs. 29, 30, and 37.
4. Consider the Young’s modulus from Eq. 38, $E\left(\rho \left(t\right),d\left(\tau \right)\right)$ , such that *ρ*(*t*) is a constant for the instant under study, *t*, but *d*(*τ*) varies throughout the integration.
5. Start with an estimation of the critical load *σ* = 0.
6. Assuming a uniaxial stress state, calculate the maximum tensile strain:

$$\varepsilon_{max}=\left\{\begin{array}{cc} \frac{\sigma }{E\left(\rho \left(t\right),d\left(\tau \right)\right)}\; & if\;\sigma \geq 0\\ -\frac{\nu \;\sigma }{E\left(\rho \left(t\right),d\left(\tau \right)\right)}\; & if\;\sigma <0 \end{array}\right.$$ (41)

7. Integrate Eq. 19 over time *τ* in the domain *τ* ∈ [*t*, *t* + 10] days. This is done with the help of Eqs. 20 and 21, which, in turn, need Eqs. 22 to 25.
8. If *d* reaches failure (*d* = 0.99 in practical terms) before 10 days, establish *σ* ^*crit*^(*t*) = *σ* and exit. Else, increment *σ* and go back to step 6 to commence a new iteration.

To compare the deterioration of bone mechanical properties with the disease and after discontinuation of the treatment, the critical load at each time point was compared to the critical load at the onset of disease giving the following safety factor:

$$SF\left(t\right)=\frac{\sigma^{\mathrm{crit}}\left(t\right)}{\sigma^{\mathrm{crit}}\left(t=0\right)}$$ (42)

## Results

### Validation of Results and Comparison of the Standard and Extended Bone Cell Population Model

**FIGURE 2:**
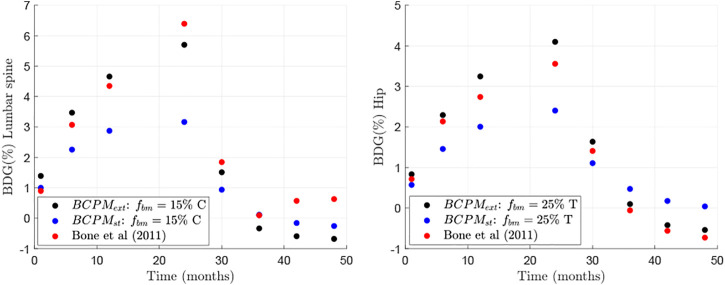
Comparison between clinical results (from Bone et al. (Bone et al., 2011)) and *in-silico* results of changes in BMD with respect to baseline for two different bone sites: lumbar spine (left) and hip (right).

The new PK-PD model proposed for Dmab (see Figure 1) was adjusted first to obtain ${\tilde{D}}_{{\text{Dmab}}_{BC}}$ and *ζ*. This was done by comparing the *in-silico* results of *BCPM*_*ext*_ with the clinical results obtained by Bone et al. (Bone et al., 2011). In the latter, the patients were subjected to a 2 years treatment of Dmab consisting in the usual dose approved by the WHO, 60 mg injections every 6 months (60Q6). The average time elapsed since menopause for those patients was around 10 years. These conditions were simulated with *BCPM*_*ext*_ for a combination of parameters. More precisely, in a 20 × 20 grid of equally spaced values defined in the range: ${\tilde{D}}_{{\text{Dmab}}_{BC}}\in \left[0,10\right]\;{day}^{-1}$ and *ζ* ∈ [0, 1]. The comparison was performed at two different sites: the lumbar spine, for which a representative porosity of 85% (f_*bm*_ = 15%) was chosen and a uniaxial compression state was assumed (*σ* = 0.15 MPa), and the hip, for which f_*bm*_ = 25% was chosen and a uniaxial tension state was simulated (*σ* = 0.5 MPa). The latter stress state would approximate the area of the femoral neck subjected to tensile stresses due to bending. The value of the stresses were adjusted to produce a homeostasis state for the corresponding value of f_*bm*_, as done in Figure 4 of (Martínez-Reina et al., 2021b). The following error was computed from the comparison:

$$E=\underset{i}{\sum }\underset{j}{\sum }{\left(BDG_{ij}^{\mathrm{model}}-BDG_{ij}^{\mathrm{clin}}\right)}^{2}$$ (43)

where the superscripts *model* and *clin* refer to the *in-silico* and clinical results, respectively; the subscript *i* stands for lumbar spine and hip and the subscript *j* refers to the time points for which the results by Bone et al. (Bone et al., 2011) were available and could be compared (see Figure 2). This error was computed for all the points of the aforementioned grid and the subregion where the error was lower was refined to search for a global minimum of *E*, obtained for the pair of values $\left({\tilde{D}}_{{\text{Dmab}}_{BC}},\zeta \right)=\left(24.5\;{day}^{-1},0.85\right)$ , which we adopted for the rest of simulations. Figure 2 compares the *in-silico* and clinical BDG results corresponding to that pair of values. First of all, it can be seen that the predictions of *BCPM*_*st*_ (blue) are far from the clinical results as the parameters ${\tilde{D}}_{{\text{Dmab}}_{BC}}$ and *ζ* were fitted for *BCPM*_*ext*_. Thus, focusing on the results obtained with *BCPM*_*ext*_ it can be seen that BDG is greater in the lumbar spine than in the femoral neck and that bone loss is very acute after the discontinuation of the treatment. This rebound of bone loss is similar at both bone sites, though it appears to stabilise in the lumbar spine, as opposed to the femoral neck.

**FIGURE 3:**
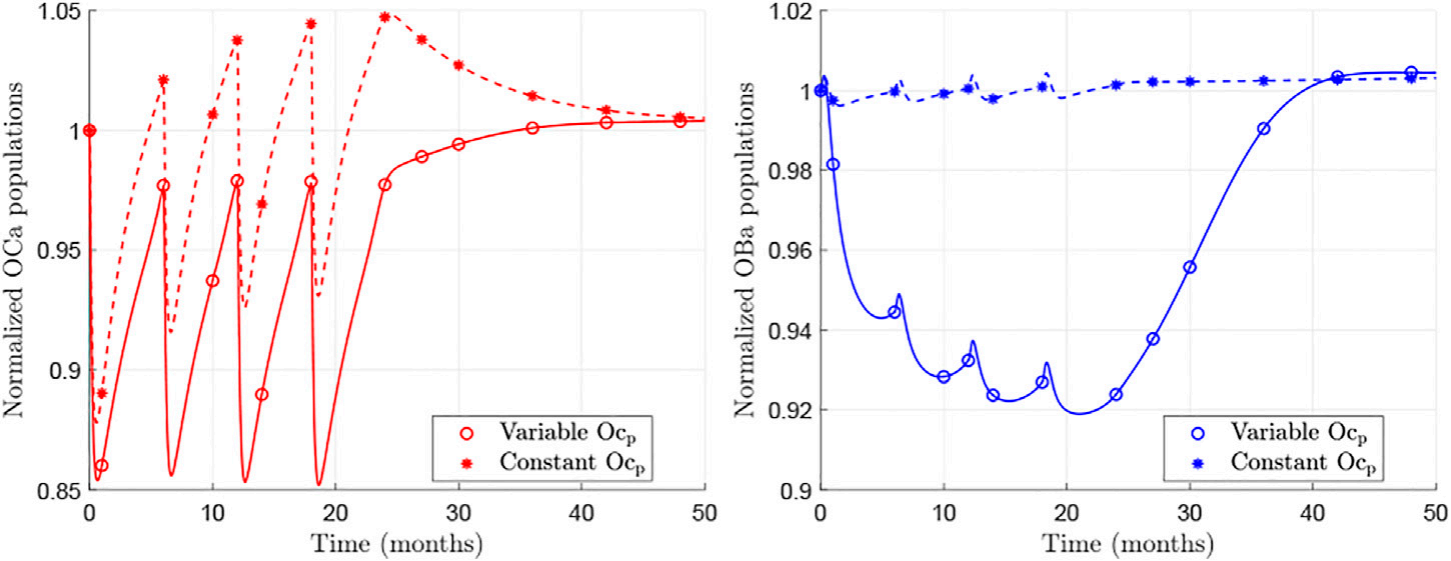
Normalised evolution of active osteoclasts (left) and active osteoblasts (right) obtained with *BCPM*_*ext*_ (solid) and *BCPM*_*st*_ (dashed). The lines represent the continuous evolutions predicted by the models and the asterisks mark the values at those time points analysed in the clinical study by Bone et al. (Bone et al., 2011).

Next we investigate the cause of the BDG rebound behaviour, which is due to the underlying changes of bone cell numbers in the BCPM. Figure 3 shows the temporal evolution of osteoclasts and osteoblasts populations for the case of the hip during Dmab treatment, normalised by the cell numbers at the beginning of the treatment. The models *BCPM*_*st*_ (dashed) and *BCPM*_*ext*_ (solid) are compared.

Focusing on the results of *BCPM*_*ext*_, one can see that the predicted population of osteoclasts falls with Dmab injections, as the drug suppresses the effect of RANKL, but as the concentration of Dmab decreases, a rebound in osteoclastic numbers is observed. When the treatment is discontinued, this rebound is prolonged and pre-treatment values are reached.

The osteoblasts population is also reduced and a rebound is observed as well. The changes in osteoblast numbers follow those of osteoclastic cells though slower and deferred in time. Consequently, the rebound is noticeable some months after stopping the Dmab treatment.

On the other hand, the behaviour predicted by the *BCPM*_*st*_ is significantly different: no rebound in osteoclastic cell numbers can be observed. On the contrary, a decrease after discontinuation of the treatment can be seen, and there are no evident changes in osteoblastic cell numbers.

**FIGURE 4:**
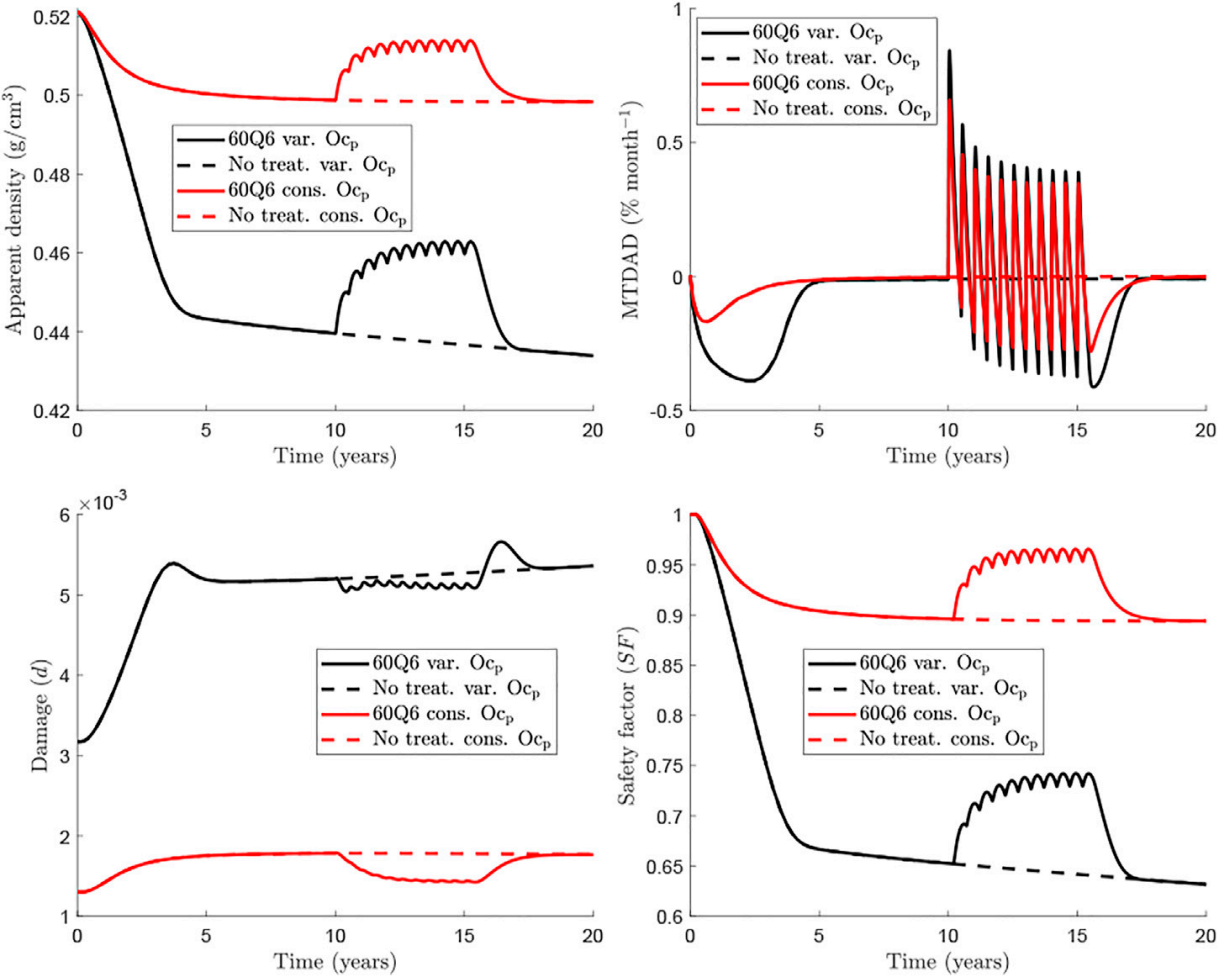
Comparison of *BCPM*_*ext*_ (black) and *BCPM*_*st*_ (red) models for the femoral neck (*f*_*bm*_ =25%). The treatment 60Q6 (solid) is compared with the no treatment case (dashed) for each model. The evolution of apparent density (top left), *MTDAD* (top right), damage (bottom left) and safety factor (bottom right) are represented.

Figure 4 compares further the predictions of the two models with respect to the evolution of apparent density (top left), *MTDAD* (top right), damage level (bottom left) and the safety factor (bottom right), which are presented for the case *f*_*bm*_ = 25% subjected to uniaxial tension and under a treatment 60Q6 beginning 10 years after menopause and discontinued after 5 years of treatment. It can be seen that menopause produces an immediate and pronounced bone loss (apparent density falls and *MTDAD* is negative) after menopause, when RANKL levels increase. Bone mass gain is evident after the Dmab injection, when the increase of RANKL levels is counteracted by the drug. Later, a series of cycles occur during the treatment and bone mass gain slows down as Dmab is cleared away from blood to lead again to bone loss at the end of each treatment cycle.

This global behaviour is seen both in *BCPM*_*ext*_ and *BCPM*_*st*_, but there is a clear difference between them, which is more evident in *MTDAD*: bone loss predicted after discontinuation by *BCPM*_*ext*_ (black line) is more pronounced and prolonged than that predicted by *BCPM*_*st*_ (red line). The risk of treatment discontinuation is quite evident looking at the evolution of damage, which presents a peak some months after the last Dmab injection, a peak which is only predicted by *BCPM*_*ext*_.

The safety factor is a variable that measures how far is the tissue to local failure and is mainly influenced by the damage level and the magnitude of strain. The latter is, in turn, influenced by the stiffness and thus by the apparent density. In summary, the safety factor is controlled by the apparent density and the damage level. However, in view of the results, it appears that the former has more influence, for such values of damage and *SF* shows a similar trend to apparent density though it decreases by 35% in 20 years while apparent density decreases only by 16% in the case shown in Figure 4.

### Influence of Body Weight

**FIGURE 5:**
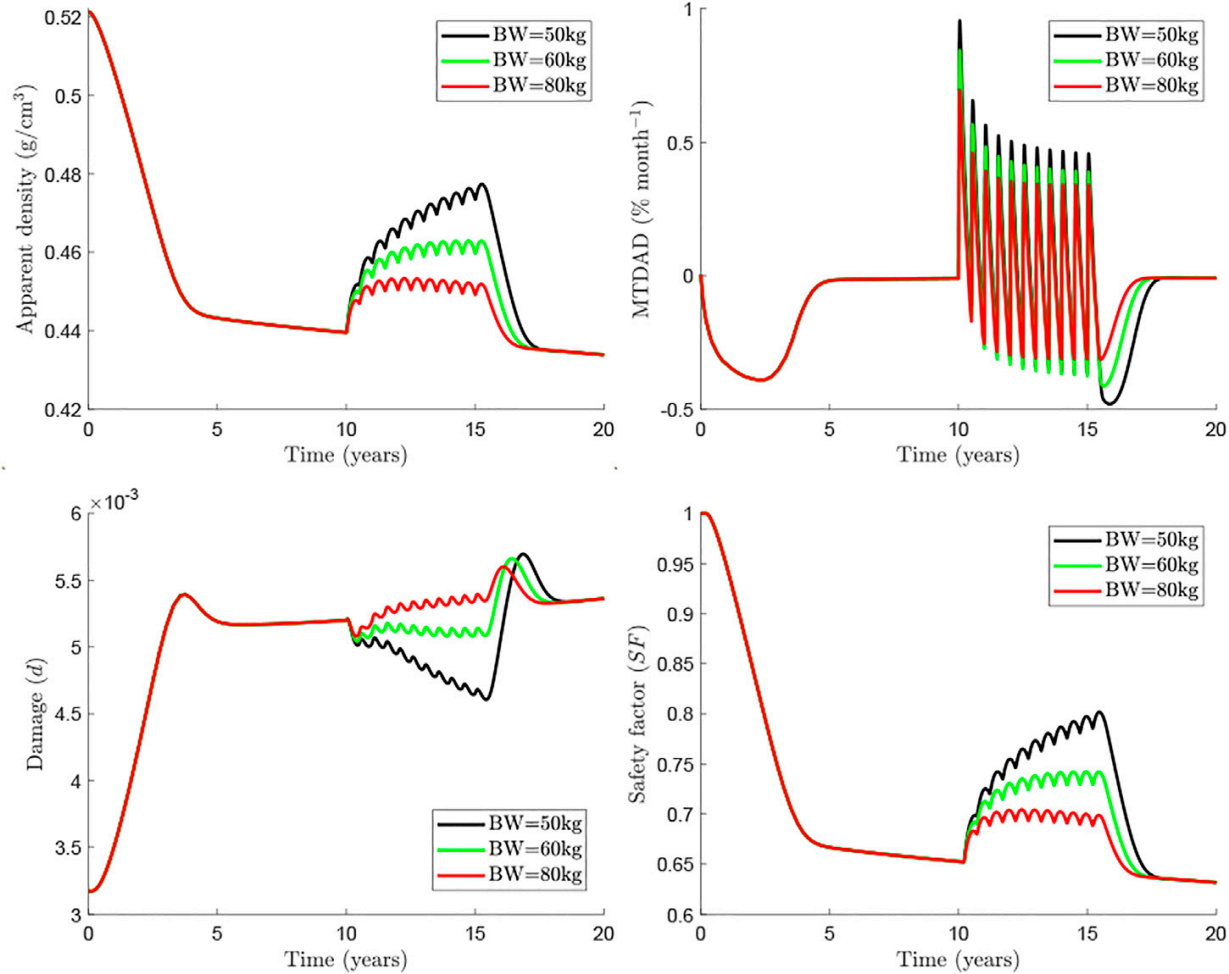
Influence of body weight in the treatment 60Q6 commencing 10 years after menopause and discontinued after 8 years.

Currently, Dmab is administered at a constant dose of 60 mg every 6 months. Consequently, dose is not adjusted for the patient’s body weight (BW) though it influences the concentration of drug present in the central compartment. Marathe et al. (Marathe et al., 2011) showed a strong influence of BW on Dmab efficacy. For this reason, a BW = 60 kg has been taken as the reference in the previous simulations. In the following, we analyse the effect of BW on the response of bone to the usual Dmab treatment 60Q6 (see Figure 5). It can be seen that the rebound in osteoclastic cell numbers seems more pronounced for lighter patients (see both apparent density and *MTDAD* plot, Figure 5). Furthermore, the increment in damage after discontinuation follows a similar trend. Nonetheless, bone density gain and consequently the safety factor, are clearly lower for heavier patients, for whom the dose of 60 mg appears to be insufficient.

**FIGURE 6:**
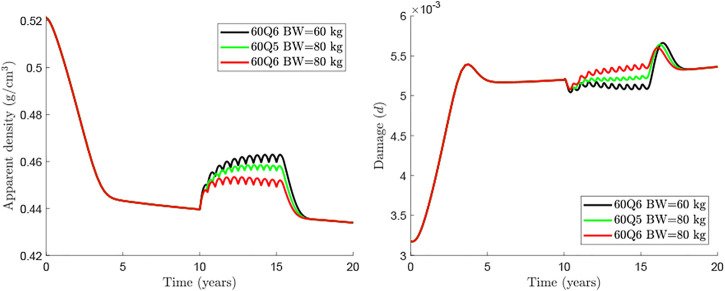
Comparison of treatments 60Q6 and 60Q5 commencing 10 years after menopause and discontinued after 8 years for different body weights.

An alternative for heavier patients could be an adjustment of the dosage, either with a greater dose or with a more frequent injection. The rationale for this option is based on the clinical results obtained by Marathe et al. (Marathe et al., 2011) on the temporal evolution of Dmab concentration in the central compartment. These authors observed a sharp drop around 5 months after the injection of 1 mg/kg of BW. This could justify the more frequent dosage in heavier patients, so to limit the bone loss rebound seen at the end of each injection cycle. Figure 6 compares the treatment 60Q5 for a patient of BW = 80 kg, which yields a quite similar response to that of the WHO-approved 60Q6 for BW = 60 kg.

### Dmab Discontinuation Strategies

Given that an abrupt withdrawal of Dmab treatment leads to rapid bone loss and increases bone fracture risk, an important question remains to be answered: “How can one safely discontinue Dmab treatment”? In this section we perform *in-silico* simulations to address this question.

**FIGURE 7:**
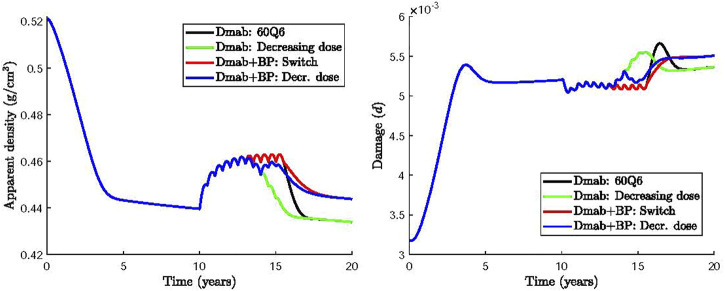
Effect of Dmab discontinuation strategies on bone apparent density and damage: 1) 60Q6 Dmab treatment discontinued after 5 years (black), 2) 60Q6 Dmab treatment with a decreasing dose (50, 40, 30, 20 and 10 mg) for the last 5 injections (green), 3) 60Q6 Dmab treatment switched to BP after 5 years (red), 4) 60Q6 Dmab treatment with a decreasing dose (50, 40, 30, 20 and 10 mg) for the last 5 injections and combined with BP from dose 30 mg onwards (blue).

One strategy would be to define a time period where Dmab is reduced to zero dose and then create a transition from the original dose (60 mg) to 0 mg by incrementally reducing the dose. Here, we simulate this transitional treatment by maintaining the frequency at 6 months and progressively decreasing the Dmab dose in the last 5 injections to 50, 40, 30, 20 and 10 mg. Figure 7 compares the 60Q6 treatment (black line) with the decreasing dose treatment (green line). It can be seen that there is no significant difference between the finally attained values for apparent bone density and damage. However, there is some difference on how quick bone is lost with the decreasing dose treatment showing a slower bone loss compared to an abrupt withdrawal. Similar, the damage variable increases more rapidly with the abrupt withdrawal compared with the decreasing dose strategy.

Several clinical studies have investigated an alternative Dmab discontinuation strategy which uses a bisphosphonate (BP) prior to or at the time for Dmab withdrawal (Kendler et al., 2020; Burckhardt et al., 2021; Tsourdi et al., 2021). BP have been reported to induce apoptosis of active osteoclasts (Halasy-Nagy et al., 2001; Takagi et al., 2021). Here we simulate the effect of BP treatment by increasing the apoptosis rate of active osteoclasts, $A_{{\text{Oc}}_{a}}$ , by 10%.

Based on these clinical studies, two other alternative treatments were considered and compared in Figure 7: 1) the intake of BP 6 months after completing the 60Q6 constant dose Dmab treatment, as recommended by (Tsourdi et al., 2021) (Dmab + BP: Switch, red line); 2) the intake of BP 48 months after the Dmab treatment has commenced, or equivalently, 12 months after the Dmab dose was decreased following the pattern described above (i.e., 50, 40, 30, 20 and 10 mg for the last 5 injections) (Dmab + BP: Decr. dose, blue line).

It can be seen that the use of BP avoids the rapid bone loss and the increase of damage compared to the abrupt withdrawal of Dmab. Same values of apparent bone density and damage are obtained independent of the time of BP administration. The incremental reduction of Dmab does not appear to have a significant benefit compared to the instantaneous Dmab discontinuation.

## Discussion

The BDG results of the model presented in this work were compared to those of clinical studies investigating the effect of drug discontinuation of Dmab treatment (Bone et al., 2011) and demonstrated good agreement (Figure 2). The only exception was the part of the BDG results for the lumbar spine for the time period after Dmab discontinuation (see Figure 2 left). In this period, Bone et al. (Bone et al., 2011) obtained a decline in bone mass up to 1 year after discontinuation followed by an increase in bone mass, a trend that could not be explained by the current model. A plausible explanation for this increase could be that patients had started another PMO treatment, not considered in the simulations. In fact, Bone et al. (Bone et al., 2011) explicitly stated: “During the off-treatment phase, if the study investigator determined that the overall fracture risk of a participant required additional treatment for osteoporosis, they could treat the participant with an approved therapy for osteoporosis”. In any case, a strong rebound in bone loss after Dmab discontinuation is observed in both *in-silico* and clinical results, in agreement with other clinical results (Zanchetta et al., 2018).

The rebound in bone loss can be explained by bone cellular activities, which are reflected in the BTMs. Bone et al. (Bone et al., 2011) measured the serum C-terminal telopeptide of type 1 collagen (sCTXI) and the N-terminal propeptide of type 1 procollagen (PINP), which correlate, respectively, with the osteoclastic and osteoblastic activity. Some authors who have implemented similar BCPMs have proposed mathematical equations to relate the concentrations of osteoclasts and osteoblasts with BTMs (Marathe et al., 2011; Post et al., 2013; Lien et al., 2020). However, use of these formulae is problematic. Indeed, BCPMs are usually developed locally to model a bone-specific response which requires definition of selected RVE parameters for specific bone sites such as vertebrae or hip. These BCPMs allow to calculate local changes in BMD and bone cell numbers. On the other hand, the aforementioned BTMs are systemic quantities (i.e. not bone-site specific) that are measured in the serum or urine. They account for the response of the whole skeleton, reflecting systemic cellular activity. For that reason, the use of such mathematical formulations of BTMs has been dismissed in the current work. Comparison between the “systemic” clinical results of BTMs and the “bone-site specific” bone cell population results is provided in the next paragraph in a qualitative way.

Figure 3 (solid lines) shows the *in-silico* results of the new model, *BCPM*_*ext*_. During the treatment osteoclast numbers oscillate, but on average decrease in concentration. After Dmab discontinuation the number of osteoclasts returns to pre-treatment levels. The clinical results obtained by Bone et al. (Bone et al., 2011) show a steep descent in sCTXI levels during the treatment, with oscillations that are smaller than those of the osteoclast concentration in the *in-silico* results and a rebound which reaches its maximum at 12 months after the last Dmab dose. *BCPM*_*ext*_ does not produce an overshoot (above baseline) in Oc_*a*_ population, but a recovery to baseline levels. However, *BCPM*_*ext*_ response is more similar to clinical results compared with the standard *BCPM*_*st*_, as the latter produces oscillations in Oc_*a*_ numbers whose peaks are too high and increase over time. Furthermore, after Dmab discontinuation *BCPM*_*st*_ predicts a decline in osteoclastic activity (Figure 3, dashed lines) which is in contrast to the clinical data. In the next paragraph we analyse the mechanisms responsible for the different model responses.

Both models realistically capture the increase of Oc_*a*_ concentration at the end of each treatment cycle when Dmab levels decay. The main difference between the two models is that *BCPM*_*st*_ considers a constant pool of Oc_*p*_, which is variable in *BCPM*_*ext*_. In the latter model, the population of Oc_*p*_ falls during the treatment, when the effect of RANKL is attenuated by Dmab. The lower concentration of precursor cells results in Oc_*a*_ values below those predicted by *BCPM*_*st*_ (Figure 3, left). On the other hand, since the differentiation of Oc_*p*_ into Oc_*a*_ was assumed to be inhibited (to a great extent in *BCPM*_*ext*_ compared to *BCPM*_*st*_) by Dmab, Oc_*p*_ are accumulated at the end of each treatment cycle in *BCPM*_*ext*_. Thus, after discontinuation both effects are superposed in *BCPM*_*ext*_: an increased differentiation rate of Oc_*p*_ into Oc_*a*_, due to clearance of Dmab, and the high concentration of Oc_*p*_ accumulated during the last treatment cycle.

McDonald et al. (McDonald et al., 2021) have recently shown that active osteoclasts do not only undergo apoptosis, but can also differentiate into another cell population. These authors stimulated the differentiation of osteoclasts in mice with soluble RANKL (sRANKL) and observed that osteoclast precursors underwent cell fusion to form active osteoclasts but also fissioned into mononucleated daughter cells, which they termed osteomorphs. RANKL inhibition through OPG resulted in the accumulation of osteomorphs and OPG withdrawal in the recycling of osteomorphs that fused to form functional osteoclasts. The role of Dmab is similar to that of OPG and it is likely that a similar phenomenon could occur during Dmab treatment of PMO. Although we have not modelled the above described mechanism, the variable pool of Oc_*p*_ can partly account for the increase of osteoclast population in *BCPM*_*ext*_.

The behaviour of osteoblasts (Figure 3 right, solid line) is similar and comparable to the clinical results, showing a decline in their activity during Dmab treatment and a recovery after discontinuation, which occurs more gradually than for osteoclasts. This behaviour is also seen in the clinical results (Bone et al., 2011): 9 months after the last Dmab dose sCTXI has increased by 30% over baseline levels, while the osteoblastic marker PINP has only returned to the baseline level.

This indicates that the increase in Ob_*a*_ population occurs delayed to that of Oc_*a*_, as predicted by *BCPM*_*ext*_. In the model, this delayed response is mediated via TGF-*β*, which is released from the bone matrix during bone resorption. TGF-*β* is known to upregulate differentiation of Ob_*u*_ into Ob_*p*_ and downregulate differentiation of Ob_*p*_ into Ob_*a*_. This temporary imbalance between osteoblastic and osteoclastic activity in favour of the latter could also explain the rebound in loss of bone mass observed after Dmab discontinuation. However, the latter interpretation needs caution as one links systemic variables (i.e. BTMs) and bone-site specific variables (i.e. BMD). Besides, the lower Oc_*a*_ numbers predicted by *BCPM*_*ext*_, on average would produce a lower release of TGF-*β* from bone matrix, with even lower numbers during Dmab treatment. This would result in a lower concentration of Ob_*p*_ and ultimately of Ob_*a*_. On the contrary, the average concentration of TGF-*β* predicted by *BCPM*_*st*_ would be higher and its effect on the populations of Ob_*p*_ and Ob_*a*_ less affected by its fluctuations, so yielding an approximately constant evolution of the number of osteoblasts, which is contrary to clinical results.

Our model also provides information on the rate of change in bone density measured by the monthly time derivative of apparent density (*MTDAD*) and the safety factor (*SF*) which is linked to how far a particular bone site is from failure. The evolution of apparent density exhibits a rapid decrease as shown by the evolution of *MTDAD*. After Dmab discontinuation *MTDAD* reaches values that are slightly greater than those predicted for the worst pre-treatment stages of the disease. Furthermore, the comparison between *BCPM*_*ext*_ and *BCPM*_*st*_ (red vs black lines in Figure 4) reinforces the fact that traditional BCPM are not able to capture the spike of bone loss, as *BCPM*_*st*_ predicts a gradual loss, with *MTDAD* being not as negative and as prolonged as predicted by *BCPM*_*ext*_.

Another interesting feature predicted by the *BCPM*_*ext*_ is a spike in microstructural damage starting around 1 year after the last Dmab dose and lasting 2–3 years (Figure 4, damage variable plot). Again, standard BCPM are not able to predict this behaviour. We note that this spike corresponds to clinical observations of atypical fractures (AFs), occurring a few months after the effect of the last Dmab injection (Anastasilakis et al., 2017). For a 60 mg Dmab dose AFs occur about 8 months after the last injection (Marathe et al., 2011).

The risk of bone failure can be estimated by analysing the evolution of the safety factor *SF* (Figure 4). *SF* depends mainly on the damage accumulated in the bone matrix and the apparent bone density, but the latter seems to have a major influence, given that the evolutions of *SF* and apparent density are very similar. However, there is a key difference: the decrease in apparent density throughout the disease or after Dmab discontinuation is magnified in the safety factor. For instance, *BCPM*_*ext*_ predicted that *SF* has decreased by 35% at the beginning of the treatment (year 10), while apparent density has decreased only by 16%. After Dmab discontinuation *SF* falls, now by 10%; while apparent density decreases only by 5%. Again, *BCPM*_*ext*_ performs better than *BCPM*_*st*_ in predicting the risk of failure after Dmab discontinuation. Thus, the former predicts a 10% drop in *SF* in about 1.5 years, while *BCPM*_*st*_ predicts a more gradual drop, by 7.5% in 2.5 years.

The influence of patient’s BW and different Dmab doses were analysed respectively in Figures 5, 6. In general, if the Dmab dosage (mg/kg of BW) is increased, BMD gain is higher, but bone loss and the rebound of damage observed after Dmab withdrawal are also more pronounced. In other words, Dmab discontinuation is potentially more dangerous in patients with low BW. However, the benefits of the drug are more evident during the treatment and this is what should prevail, since the long-term response appears to be independent of BW. Thus, the *in-silico* results would indicate that the dosage 60Q6 could be suboptimal for an 80 kg patient given that bone gain is only moderate during the treatment and the lower rebound of damage after discontinuation would not justify its use since it is only slightly lower than in patients with low BW. On the other hand, decreasing the time between injections to 5 months (60Q5) has beneficial effects on bone density as Dmab is not totally depleted between injections (Marathe et al., 2011) and the spike of damage after Dmab withdrawal is not much higher than with 60Q6.

Motivated by the adverse effects of Dmab discontinuation for the dosage 60Q6 some alternative treatments were investigated. A gradual decrease in the dose during the last years of the treatment had no significant benefits as bone density loss is more pronounced and the spike of damage is only slightly reduced. On the other hand, administration of a different drug that promotes osteoclasts apoptosis, such as BP, can alleviate bone loss (Figure 7). Both switching from Dmab to BP (red line) or administering BP concurrently while the Dmab dose is being reduced (blue line), lead to significant reduction of BMD loss after Dmab withdrawal. The predicted values of microstructural damage are higher in the Dmab + BP cases, as the lower bone turnover rate is not able to repair bone matrix as effectively as in the Dmab cases. However, as mentioned above, apparent density has more impact on the safety factor than damage, at least for the levels reached in these simulations, and thus *SF* is eventually higher in the Dmab + BP cases. The evolution of *SF* is not shown in Figure 7 as it is almost identical to the evolution of apparent density though with different values. Thus, while *SF* falls by 37% in the Dmab cases after 20 years (10 years after the onset of the disease), it only falls by 33% in the Dmab + BP case. This would be in accordance with the retrospective study by Burckhardt et al. (Burckhardt et al., 2021) who found that a treatment with BP, especially after Dmab, had a protective effect. On the other hand, it was found that the final values of BMD and damage are not sensitive to the time of administration of BP.

We finally want to point out some limitations of our *in-silico* results. The effect of BP has been considered in the model in a simplistic way, by assuming that BP increases the apoptosis rate of osteoclasts by a fixed value that remains constant over time. In the future, a more detailed model describing the pharmacokinetics (PK) of BP must be developed. That PK model would be tailored to a particular BP such as alendronate. Other possible actions of BP on bone cells might need to be taken into account in this model. It has been reported that certain BP limit the resorbing capacity of osteoclasts (Halasy-Nagy et al., 2001; Takagi et al., 2021); and finally an important aspect which is specific to BP, the fact that it may remain trapped within bone matrix for long periods of time, until it is released through bone resorption. These aspects require further model refinements and are out of scope of the current paper.

The case assumed here to simulate the behaviour of the lumbar spine was a RVE of *f*_*bm*_ = 15% subjected to uniaxial compression, while *f*_*bm*_ = 25% and uniaxial tension was assumed for the hip. The latter would approximate the behaviour of the trabecular region of the femoral neck subjected to bending. Both assumptions constitute an important limitation of the study and are justified by the use of a RVE to implement the model. However, an organ level finite element (FE) model is needed to study bone strength and fracture risk in a more realistic manner.

## Summary and Conclusions

In this paper, a previously published bone cell population model (*BCPM*_*st*_) has been extended (*BCPM*_*ext*_) to simulate the rebound of bone loss observed after discontinuation of Dmab treatment in women with post-menopausal osteoporosis. The main difference between both models is that *BCPM*_*ext*_ considers a variable pool of osteoclast precursors, which were considered constant in the previous one. *BCPM*_*ext*_ predicts that these precursors accumulate at the end of each treatment cycle. Thus, once Dmab is depleted and RANKL is restored to pre-treatment values, the differentiation rate of precursors into mature osteoclasts tends to rise, as at the end of each treatment cycle, but the larger amount of available precursors enhance this phenomenon and that differentiation rate even exceeds pre-treatment values. This spike of bone loss induces a rebound in microstructural damage and these two factors together may be responsible for the atypical fractures reported in numerous clinical studies.

The *in-silico* results allow to draw the following conclusions:

• The accumulation of osteoclast precursors (or possibly osteomorphs) could explain the spike in bone loss after Dmab discontinuation.
• The adjustment of Dmab dose to body weight needs to be explored since the WHO-approved dose (60 mg injected every 6 months) could be insufficient for patients with high BW.
• Gradually decreasing the Dmab dose during the last years of treatment as an alternative to the abrupt discontinuation showed no clear benefits, but a transition to other drugs with different effects on bone turnover, such as BP that increase osteoclast apoptosis rate, might be a good strategy to reduce the fracture risk observed after Dmab discontinuation.
• The long-term values of BMD and damage are not sensitive to the time of administration of the BP in combined treatments.

These conclusions must be confirmed in future studies, both clinical and *in-silico*. The latter have the advantage that many combinations can be tested in a quick and systematic way, but they require the application of the BCPM in a FE model that allows considering different bone structural aspects not taken into account here. These aspects include more realistic loads, the influence of heterogeneity on bone density changes, microstructural damage accumulation and stress redistribution.

## Data Availability

The raw data supporting the conclusions of this article will be made available by the authors, without undue reservation.

## Footnotes

1 In the damage model proposed by (Martínez-Reina et al., 2009), cracks were assumed to grow normal to the maximum strain direction and only under tensile strains, if *ɛ*_*max*_ > 0. For example, in the case of uniaxial compression these strains are due to the Poisson's effect.
