# Supplementary material for: Assessment of Strategies for Safe Drug Discontinuation and Transition of Denosumab Treatment in PMO—Insights From a Mechanistic PK/PD Model of Bone Turnover
Source: Front Bioeng Biotechnol. 2022 Jun 17;10:886579. doi: 10.3389/fbioe.2022.886579 (PMC9367195; doi:10.3389/fbioe.2022.886579)
Supplement: Supplementary file 1 [file DataSheet1.pdf]

Supplementary Material to: Assessment of strategies for safe drug discontinuation and transition of denosumab treatment in PMO – Insights from a mechanistic PK/PD model of bone turnover

Javier Martínez-Reina[a,1,*], José Luis Calvo Gallego[a,1], Madge Martin[b,1], Peter Pivonka[c,1]

[a] *Departmento de Ingeniería Mecánica y Fabricación, Universidad de Sevilla, Seville 41092, Spain.*

[b] *Laboratoire Modélisation et Simulation Multi-Echelle (MSME), UMR CNRS 8208, Université Paris-Est Créteil, 61 Avenue du Général de Gaulle, Créteil 94010, France.*

[c] *School of Mechanical, Medical and Process Engineering, Queensland University of Technology, QLD 4000, Australia.*

1. Introduction

In this document we provide those details of the mathematical model not provided in the main document. More precisely, we explain here those features of the original model by Martin et al. [1] which remained unchanged; while those that have been modified or are more relevant to the study are presented in the main document.

This document is structured as follows: In section 2 we present the general equations for competitive binding between ligands and receptors; the specific equations for the competitive binding of the complex Wnt–Scl–LRP5/6 are given in section 3 and the co-regulation of RANKL levels via the antagonistic effect of PTH and nitric oxide in section 4. The equations for the competitive binding of the complex RANK-RANKL-OPG-Dmab were given in the main document. The upregulation of RANKL expressed by osteocytes due to damage is given in section 5. The equations related to the regulatory effect of TGF-β are given in section 6. Section 7 describes the term of proliferation of osteoblast precursors. Finally, the model constants are given in Table 1.

*Corresponding author

2. Competitive binding

Many biological processes are controlled by binding of biochemical factors which act as receptor and ligand. In some of them two or more ligands compete to bind to the receptor. This is the case of Wnt and sclerostin that compete to bind to LRP5/6 to control the proliferation of osteoblast precursors and also the case of RANK, OPG and Dmab which compete to bind to RANKL to control the differentiation of osteoclast precursors into mature active osteoclasts.

Let us consider separately the binding of a given receptor R to its ligands A and B to form, respectively, the complexes A–R and B–R. Let us consider for each species X=A,B,R a production term P_{X} and a degradation term D_{X}, along with a degradation term for the complex $D_{\mathrm{X-Y}}$. Let $K^{r}_{\mathrm{X-Y}}$ and $K^{f}_{\mathrm{X-Y}}$ be the reverse and forward binding reaction constants, respectively.

$$\begin{array}{ccccccc} \downarrow P_{\mathrm{R}} & & \downarrow P_{\mathrm{A}} & & & & \\ & & & K^{f}_{\mathrm{A-R}} & & D_{\mathrm{A-R}} & \\ \mathrm{R} & + & \mathrm{A} & \rightleftharpoons & \mathrm{A-R} & \rightharpoonup & \emptyset \\ & & & K^{r}_{\mathrm{A-R}} & & & \\ \downarrow D_{\mathrm{R}} & & \downarrow D_{\mathrm{A}} & & & & \end{array} \tag{1}$$

$$\begin{array}{ccccccc} \downarrow P_{\mathrm{R}} & & \downarrow P_{\mathrm{B}} & & & & \\ & & & K^{f}_{\mathrm{B-R}} & & D_{\mathrm{B-R}} & \\ \mathrm{R} & + & \mathrm{B} & \rightleftharpoons & \mathrm{B-R} & \rightharpoonup & \emptyset \\ & & & K^{r}_{\mathrm{B-R}} & & & \\ \downarrow D_{\mathrm{R}} & & \downarrow D_{\mathrm{B}} & & & & \end{array} \tag{2}$$

The law of mass action provides the following set of differential equations:

$$\frac{\partial[\mathrm{A-R}]}{\partial t} = K^{f}_{\mathrm{A-R}}\,[\mathrm{A}]\,[\mathrm{R}] - K^{r}_{\mathrm{A-R}}\,[\mathrm{A-R}] - \tilde{D}_{\mathrm{A-R}}\,[\mathrm{A-R}] \tag{3a}$$

$$\frac{\partial[\mathrm{B-R}]}{\partial t} = K^{f}_{\mathrm{B-R}}\,[\mathrm{B}]\,[\mathrm{R}] - K^{r}_{\mathrm{B-R}}\,[\mathrm{B-R}] - \tilde{D}_{\mathrm{B-R}}\,[\mathrm{B-R}] \tag{3b}$$

$$\frac{\partial[\mathrm{A}]}{\partial t} = P_{\mathrm{A}} - \tilde{D}_{\mathrm{A}}\,[\mathrm{A}] + K^{r}_{\mathrm{A-R}}\,[\mathrm{A-R}] - K^{f}_{\mathrm{A-R}}\,[\mathrm{A}]\,[\mathrm{R}] \tag{3c}$$

$$\frac{\partial[\mathrm{B}]}{\partial t} = P_{\mathrm{B}} - \tilde{D}_{\mathrm{B}}\,[\mathrm{B}] + K^{r}_{\mathrm{B-R}}\,[\mathrm{B-R}] - K^{f}_{\mathrm{B-R}}\,[\mathrm{B}]\,[\mathrm{R}] \tag{3d}$$

$$\frac{\partial[\mathrm{R}]}{\partial t} = P_{\mathrm{R}} - \tilde{D}_{\mathrm{R}}[\mathrm{R}] + K^{r}_{\mathrm{A-R}}[\mathrm{A-R}] + K^{r}_{\mathrm{B-R}}[\mathrm{B-R}] - K^{f}_{\mathrm{A-R}}[\mathrm{A}][\mathrm{R}] - K^{f}_{\mathrm{B-R}}[\mathrm{B}][\mathrm{R}] \tag{3e}$$

where $[\mathrm{X}]$ and $[\mathrm{X-Y}]$ represent, respectively, the concentration of species X and complex X-Y. The degradation terms are assumed proportional to the concentration of the species, i.e. $D_{\mathrm{X}} = \tilde{D}_{\mathrm{X}}[\mathrm{X}]$, with $\tilde{D}_{\mathrm{X}}$ being the degradation rate.

Following Pivonka et al. [2] we assume that the binding reactions are much faster than the cell responses they produce and hence a quasi-steady state can be assumed, implying that the time derivatives of Eqs. (3) are null. This condition in Eqs. (3a) and (3b) yield:

$$[\mathrm{A-R}] = \frac{[\mathrm{A}][\mathrm{R}]}{K_{\mathrm{A-R}}} \tag{4a}$$

$$[\mathrm{B-R}] = \frac{[\mathrm{B}][\mathrm{R}]}{K_{\mathrm{B-R}}} \tag{4b}$$

where:

$$K_{\mathrm{A-R}} = \frac{K^{r}_{\mathrm{A-R}} + \tilde{D}_{\mathrm{A-R}}}{K^{f}_{\mathrm{A-R}}} \tag{5a}$$

$$K_{\mathrm{B-R}} = \frac{K^{r}_{\mathrm{B-R}} + \tilde{D}_{\mathrm{B-R}}}{K^{f}_{\mathrm{B-R}}} \tag{5b}$$

The stationarity condition of Eqs. (3c)-(3e) yields:

$$[\mathrm{A}] = \frac{P_{\mathrm{A}}}{\tilde{D}_{\mathrm{A}} + \frac{\tilde{D}_{\mathrm{A-R}}}{K_{\mathrm{A-R}}}[\mathrm{R}]} \tag{6a}$$

$$[\mathrm{B}] = \frac{P_{\mathrm{B}}}{\tilde{D}_{\mathrm{B}} + \frac{\tilde{D}_{\mathrm{B-R}}}{K_{\mathrm{B-R}}}[\mathrm{R}]} \tag{6b}$$

$$[\mathrm{R}] = \frac{P_{\mathrm{R}}}{\tilde{D}_{\mathrm{R}} + \frac{\tilde{D}_{\mathrm{A-R}}}{K_{\mathrm{A-R}}}[\mathrm{A}] + \frac{\tilde{D}_{\mathrm{B-R}}}{K_{\mathrm{B-R}}}[\mathrm{B}]} \tag{6c}$$

If the degradation ($\tilde{D}_{\mathrm{X}}$,$\tilde{D}_{\mathrm{X-Y}}$), production (P_{X}) and dissociation constants, ($K_{\mathrm{X-Y}}$) are known, (6) constitutes a non-linear system of three equations with three unknowns, namely [A], [B], [R].

The total concentration of a receptor is the sum of the concentrations of the receptor which is found free and bound to ligands, i.e.:

$$[\mathrm{R}]_{\mathrm{tot}} = [\mathrm{R}] + [\mathrm{A-R}] + [\mathrm{B-R}] = [\mathrm{R}]\left(1 + \frac{[\mathrm{A}]}{K_{\mathrm{A-R}}} + \frac{[\mathrm{B}]}{K_{\mathrm{B-R}}}\right) \tag{7}$$

where Eqs. (4) have been used. The stationarity condition is equivalent to establish that the production rate of a species must equal the degradation rate, including all its forms, free and bound. For instance, in the case of the receptor that can bind to different ligands, this condition reads:

$$P_{\mathrm{R}} = \tilde{D}_{\mathrm{R}}\,[\mathrm{R}] + \sum_{\mathrm{L}} \tilde{D}_{\mathrm{L-R}}\,[\mathrm{L-R}] \tag{8}$$

which can be obtained from Eqs. (3a), (3b) and (3e) by imposing that the stationarity condition is met, ($\frac{\partial[\mathrm{R}]}{\partial t} = \frac{\partial[\mathrm{L-R}]}{\partial t} = 0 \quad \forall\,\mathrm{L} = \mathrm{A},\mathrm{B}$). In the case of a ligand, that only binds to the receptor, that condition reads:

$$P_{\mathrm{L}} = \tilde{D}_{\mathrm{L}}\,[\mathrm{L}] + \tilde{D}_{\mathrm{L-R}}\,[\mathrm{L-R}] \tag{9}$$

The degradation rates are usually assumed as constants but the production rates are modelled in a more complex way. For instance, the production rate of ligand L can be split into a term corresponding to endogenous production, $P_{\mathrm{L,b}}$, and a term accounting for external dosage, $P_{\mathrm{L,d}}$:

$$P_{\mathrm{L}} = P_{\mathrm{L,b}} + P_{\mathrm{L,d}} \tag{10}$$

The endogenous production is sometimes modelled by the following equation:

$$P_{\mathrm{L,b}} = \sum_{\mathrm{X,Y}} \beta_{\mathrm{L,Y}}\,\pi^{\mathrm{X}}_{\mathrm{act/rep,Y}}\,\mathrm{Y}\left(1 - \frac{[\mathrm{L}]}{[\mathrm{L}]_{\mathrm{max}}}\right) \tag{11}$$

where Y is the concentration of the cell type Y producing L with a production rate $\beta_{\mathrm{L,Y}}$, regulated by the species X through the activator or repressor function $\pi^{\mathrm{X}}_{\mathrm{act/rep,Y}}$. The parenthesis establishes a saturation condition in such a way that ligand is not produced if its concentration reaches the maximum or saturation value, $[\mathrm{L}]_{\mathrm{max}}$.

As described by Pivonka et al. [3] the activation of a certain biological process regulated by the formation of the complex L–R is given by the ratio between the receptors R occupied by ligands L and the total number of receptors:

$$\pi^{\mathrm{L}}_{\mathrm{act,Y}} = \frac{[\mathrm{L}-\mathrm{R}]}{[\mathrm{R}]_{\mathrm{tot}}} = \frac{[\mathrm{L}-\mathrm{R}]}{[\mathrm{R}] + \sum_{\mathrm{L}'} [\mathrm{L}'-\mathrm{R}]} \tag{12}$$

Similarly, the repressor action of the binding is given by the complementary to one of the latter:

$$\pi^{\mathrm{L}}_{\mathrm{rep,Y}} = \frac{[\mathrm{R}]_{\mathrm{tot}} - [\mathrm{L}-\mathrm{R}]}{[\mathrm{R}]_{\mathrm{tot}}} = \frac{[\mathrm{R}] + \sum_{\mathrm{L}'\neq\mathrm{L}} [\mathrm{L}'-\mathrm{R}]}{[\mathrm{R}] + \sum_{\mathrm{L}'} [\mathrm{L}'-\mathrm{R}]} \tag{13}$$

In case of a single ligand the latter expressions yield the first-order Hill activator and repressor functions:

$$\pi^{\mathrm{L}}_{\mathrm{act,Y}} = \frac{[\mathrm{L}]}{[\mathrm{L}] + K^{\mathrm{L-R}}_{\mathrm{act,Y}}} \tag{14}$$

$$\pi^{\mathrm{L}}_{\mathrm{rep,Y}} = \frac{K^{\mathrm{L-R}}_{\mathrm{rep,Y}}}{K^{\mathrm{L-R}}_{\mathrm{rep,Y}} + [\mathrm{L}]} \tag{15}$$

3. Competitive binding Wnt–Scl–LRP5/6

The previous equations can be used to describe the competitive binding Wnt–Scl–LRP5/6. Wnt signaling is an anabolic pathway promoting the proliferation of osteoblast precursors and hence bone formation. Extracellular Wnt binds to Frizzled and the lipoprotein receptor-related protein LRP5/6, so triggering intracellular activation of β-catenin. Sclerostin, produced by osteocytes, modulates the signaling pathway by its interaction

with LRP5/6 receptors. This prevents the formation of the Wnt-Frizzled-LRP5/6 complex and therefore hinders preosteoblasts proliferation. Competitive Wnt–Scl–LRP5/6 binding is modelled as follows. First, Eq. (7) reads for LRP5/6:

$$[\mathrm{LRP5/6}]_{\mathrm{tot}} = [\mathrm{LRP5/6}] \cdot \left(1 + \frac{[\mathrm{Wnt}]}{K_{\mathrm{Wnt-LRP5/6}}} + \frac{[\mathrm{Scl}]}{K_{\mathrm{Scl-LRP5/6}}}\right) \tag{16}$$

The production of sclerostin is given by an equation like (9), which now reads:

$$P_{\mathrm{Scl,b}} + P_{\mathrm{Scl,d}} = \tilde{D}_{\mathrm{Scl}}\,[\mathrm{Scl}] + \tilde{D}_{\mathrm{Scl-LRP5/6}}\,[\mathrm{Scl-LRP5/6}] \tag{17}$$

where $\tilde{D}_{\mathrm{Scl}}$ and $\tilde{D}_{\mathrm{Scl-LRP5/6}}$ are the degradation rates of sclerostin and the sclerostin-LRP5/6 complex, respectively. The concentration of this complex is given by the receptor-ligand binding equation (3a), which reads here:

$$[\mathrm{Scl-LRP5/6}] = \frac{[\mathrm{Scl}]\,[\mathrm{LRP5/6}]}{K_{\mathrm{Scl-LRP5/6}}} \tag{18}$$

The external dosage of sclerostin, $P_{\mathrm{Scl,d}}$, is set to zero and the endogenous production of sclerostin by osteocytes is:

$$P_{\mathrm{Scl,b}} = \beta_{\mathrm{Scl,Ot}}\,\pi^{\Psi_{\mathrm{bm}}}_{\mathrm{rep,Scl}}\,\mathrm{Ot}\left(1 - \frac{[\mathrm{Scl}]}{[\mathrm{Scl}]_{\mathrm{max}}}\right) \tag{19}$$

where $\beta_{Scl,Ot}$ and $[\mathrm{Scl}]_{\mathrm{max}}$ are, respectively, the sclerostin production rate and its maximum concentration. The production of sclerostin by osteocytes is downregulated by the mechanical stimulus through the repressor function $\pi^{\Psi_{\mathrm{bm}}}_{\mathrm{rep,Scl}}$ (see Eq. (36) later on). Replacing (19) and (18) into (17) yields:

$$\beta_{\mathrm{Scl,Ot}}\,\pi^{\Psi_{\mathrm{bm}}}_{\mathrm{rep,Scl}}\,\mathrm{Ot}\left(1 - \frac{[\mathrm{Scl}]}{[\mathrm{Scl}]_{\mathrm{max}}}\right) = \tilde{D}_{\mathrm{Scl}}\,[\mathrm{Scl}] + \tilde{D}_{\mathrm{Scl-LRP5/6}}\,\frac{[\mathrm{Scl}]\,[\mathrm{LRP5/6}]}{K_{\mathrm{Scl-LRP5/6}}} \tag{20}$$

Following Martin et al. [1] we assumed that the total number of LRP5/6 receptors per osteoblast precursor ($N^{\mathrm{LRP5/6}}_{\mathrm{OB_p}}$) is constant and thus:

$$[\mathrm{LRP5/6}]_{\mathrm{tot}} = N_{\mathrm{OB_p}}^{\mathrm{LRP5/6}}\, \mathrm{Ob_p} \tag{21}$$

Solving for [LRP5/6] in Eq. (16) and replacing it into (20) yields the following second-order polynomial of the free sclerostin, [Scl]:

$$A\,[\mathrm{Scl}]^2 + B\,[\mathrm{Scl}] + C = 0 \tag{22}$$

where the constants coefficients of the polynomial are:

$$A = \tilde{D}_{\mathrm{Scl}} + \frac{\beta_{\mathrm{Scl,Ot}}\, \pi_{\mathrm{rep,Scl}}^{\Psi_{\mathrm{bm}}}\, \mathrm{Ot}}{[\mathrm{Scl}]_{\max}} \tag{23}$$

$$\begin{aligned} B =& A \cdot K_{\mathrm{Scl-LRP5/6}} \left(1 + \frac{[\mathrm{Wnt}]}{K_{\mathrm{Wnt-LRP5/6}}}\right) + \tilde{D}_{\mathrm{Scl-LRP5/6}}\,[\mathrm{LRP5/6}]_{\mathrm{tot}} \\ &- (P_{\mathrm{Scl,d}} + \beta_{\mathrm{Scl,Ot}}\, \pi_{\mathrm{rep,Scl}}^{\Psi_{\mathrm{bm}}}\, \mathrm{Ot}) \end{aligned} \tag{24}$$

$$C = - K_{\mathrm{Scl-LRP5/6}} \left(1 + \frac{[\mathrm{Wnt}]}{K_{\mathrm{Wnt-LRP5/6}}}\right) (P_{\mathrm{Scl,d}} + \beta_{\mathrm{Scl,Ot}}\, \pi_{\mathrm{rep,Scl}}^{\Psi_{\mathrm{bm}}}\, \mathrm{Ot}) \tag{25}$$

$$\tag{26}$$

We can use (21) in the previous expression together with the previously calculated cell populations and [Wnt], which is assumed constant, to work out the three coefficients. Only one solution of (22) is positive as shown by Martin et al. [1] and this solution [Scl] is then used in (16) to calculate [LRP5/6]. Finally, using Eqs. (12) and (21), the activator function in the Ob_p proliferation term can be calculated as:

$$\pi_{\mathrm{act,Ob_p}}^{\mathrm{Wnt}} = \frac{[\mathrm{Wnt-LRP5/6}]}{[\mathrm{LRP5/6}]_{\mathrm{tot}}} = \frac{[\mathrm{Wnt}]\,[\mathrm{LRP5/6}]}{K_{\mathrm{Wnt-LRP5/6}}\,[\mathrm{LRP5/6}]_{\mathrm{tot}}} \tag{27}$$

Some studies have shown an increase in serum sclerostin after menopause [4, 5], while sclerostin expression (local mRNA levels) was found to decrease in animal models of menopause [5]. Following [1] we have assumed an exponential decay of the degradation rate of sclerostin to acknowledge this discrepancy between the serum levels and the local expression of sclerostin:

$$\tilde{D}_{\mathrm{Scl}}(t) = \begin{cases} \tilde{D}^0_{\mathrm{Scl}} \exp\left(\dfrac{t - t_{\mathrm{onset}}}{\tau_{PMO}}\right) & \text{for } t \geq t_{\mathrm{onset}} \\ \tilde{D}^0_{\mathrm{Scl}} & \text{for } t < t_{\mathrm{onset}} \end{cases} \tag{28}$$

where t_{onset} is the time of onset of menopause, $\tilde{D}^0_{\mathrm{Scl}}$ is the pre-menopause value of the degradation rate of sclerostin and τ_{PMO} is a time constant.

4. Co-regulation of RANKL via PTH and NO concentration

RANKL transcription is upregulated by parathyroid hormone (PTH) and downregulated by nitric oxide (NO). In the model developed by Martin et al. [1] this antagonistic influence was merged into a co-regulatory function capturing both effects.

$$\pi^{\mathrm{PTH,NO}}_{\mathrm{act/rep,RANKL}} = \lambda_s \left(\pi^{\mathrm{PTH}}_{\mathrm{act,RANKL}} + \pi^{\mathrm{NO}}_{\mathrm{rep,RANKL}}\right) + \lambda_c \, \pi^{\mathrm{PTH}}_{\mathrm{act,RANKL}} \cdot \pi^{\mathrm{NO}}_{\mathrm{rep,RANKL}} \tag{29}$$

where the activator function accounting for the effect of PTH is:

$$\pi^{\mathrm{PTH}}_{\mathrm{act,RANKL}} = \frac{[\mathrm{PTH}]}{[\mathrm{PTH}] + K^{\mathrm{PTH}}_{\mathrm{act}}} \tag{30}$$

and the repressor effect on OPG (see Eq. (10) in the main document) is accounted for through the function:

$$\pi^{\mathrm{PTH}}_{\mathrm{rep,Ob_a}} = \frac{K^{\mathrm{PTH}}_{\mathrm{rep}}}{[\mathrm{PTH}] + K^{\mathrm{PTH}}_{\mathrm{rep}}} \tag{31}$$

being $K^{\mathrm{PTH}}_{\mathrm{act}}$ and $K^{\mathrm{PTH}}_{\mathrm{rep}}$ constants and the concentration of PTH given by:

$$[\mathrm{PTH}] = \frac{\beta_{\mathrm{PTH}}}{\tilde{D}_{\mathrm{PTH}}} \tag{32}$$

which comes from Eqs. (8) to (11) when there is no ligand for the species, the external dosage is null ($P_{\mathrm{PTH,d}} = 0$), the endogenous production rate is not

regulated ($\pi^{\mathrm{X}}_{\mathrm{act/rep,Y}} \cdot \mathrm{Y} = 1$ see Eq. (11)) and the saturation value $[\mathrm{PTH}]_{\mathrm{max}}$ is large enough to assume the parenthesis equal to 1. β_{PTH} and $\tilde{D}_{\mathrm{PTH}}$ are the endogenous production and degradation rate of PTH, respectively. On the other hand, the factor corresponding to nitric oxide is:

$$\pi^{\mathrm{NO}}_{\mathrm{rep,RANKL}} = \frac{K^{\mathrm{NO}}_{\mathrm{rep}}}{[\mathrm{NO}] + K^{\mathrm{NO}}_{\mathrm{rep}}} \tag{33}$$

with $K^{\mathrm{NO}}_{\mathrm{rep}}$ a constant and the concentration of NO given by:

$$[\mathrm{NO}] = \frac{P_{\mathrm{NO,d}} + \beta_{\mathrm{NO,Ot}}\, \pi^{\Psi_{\mathrm{bm}}}_{\mathrm{act,NO}}\, \mathrm{Ot}}{\tilde{D}_{\mathrm{NO}} + \frac{\beta_{\mathrm{NO,Ot}}\, \pi^{\Psi_{\mathrm{bm}}}_{\mathrm{act,NO}}\, \mathrm{Ot}}{[\mathrm{NO}]_{\mathrm{max}}}} \tag{34}$$

which also comes from Eq. (8) in the absence of ligands. The external dosage of nitric oxide $P_{\mathrm{NO,d}}$ is set to zero in this study, $\beta_{\mathrm{NO,Ot}}$, $\tilde{D}_{\mathrm{NO}}$ and $[\mathrm{NO}]_{\mathrm{max}}$ are, respectively, the endogenous production and degradation rate of nitric oxide and its maximum content. The factor $\pi^{\Psi_{\mathrm{bm}}}_{\mathrm{act,NO}}$ is the mechanical feedback activator function that accounts for the production of NO by osteocytes. This function and the repressor function affecting the production of sclerostin by osteocytes (see Eq. (19)) are defined by the following sigmoidal functions:

$$\pi^{\Psi_{\mathrm{bm}}}_{\mathrm{act,NO}} = \rho_{\mathrm{act}} + \frac{\left(\alpha_{\mathrm{act}} - \rho_{\mathrm{act}}\right) \Psi_{\mathrm{bm}}^{\gamma^{\mathrm{act}}}}{\delta_{\mathrm{act}}^{\gamma^{\mathrm{act}}} + \Psi_{\mathrm{bm}}^{\gamma^{\mathrm{act}}}} \tag{35}$$

$$\pi^{\Psi_{\mathrm{bm}}}_{\mathrm{rep,Scl}} = \alpha_{\mathrm{rep}} - \frac{\left(\alpha_{\mathrm{rep}} - \rho_{\mathrm{rep}}\right) \Psi_{\mathrm{bm}}^{\gamma^{\mathrm{rep}}}}{\delta_{\mathrm{rep}}^{\gamma^{\mathrm{rep}}} + \Psi_{\mathrm{bm}}^{\gamma^{\mathrm{rep}}}} \tag{36}$$

where $\rho_{\sim}$ and $\alpha_{\sim}$ are, respectively, the minimum and maximum anticipated response, $\gamma^{\sim}$ is the sigmoidicity, influencing the steepness of the response, and $\delta_{\sim}$ is the value of the stimulus producing the half-maximal response [6].

Finally, the strain energy density (SED) at the bone matrix level is given by the SED at the continuum level, $\Psi = \frac{1}{2}\boldsymbol{\sigma} : \boldsymbol{\varepsilon}$, and the bone matrix fraction through the following expression proposed by Beaupré et al. [7]:

$$\Psi_{\mathrm{bm}} = \frac{\Psi}{\left(\frac{f_{\mathrm{bm}}}{100}\right)^2} \tag{37}$$

5. Upregulation of RANKL expressed by osteocytes due to microstructural damage

As proposed in [8] we have assumed that RANKL expression by osteocytes is upregulated by the presence of microstructural damage in the bone matrix through the factor $\pi^{\mathrm{dam}}_{\mathrm{act,RANKL}}$, which is defined as a sigmoidal function of damage, d:

$$\pi^{\mathrm{dam}}_{\mathrm{act,RANKL}} = \rho_{dam} + (\alpha_{dam} - \rho_{dam})\,(1 + K_{dam})\,\frac{d}{K_{dam} + d} \tag{38}$$

where K_{dam} is a constant, ρ_{dam} is the minimum value of the factor $\pi^{\mathrm{dam}}_{\mathrm{act,RANKL}}$, corresponding to $d = 0$, while α_{dam} is its maximum value, corresponding to $d = 1$.

6. Regulatory role of TGF-β

TGF–β is stored in the bone matrix and released during resorption by osteoclasts. Its concentration is calculated following Pivonka et al. [2]:

$$[\mathrm{TGF}-\beta] = \frac{\alpha_{\mathrm{TGF}-\beta}\,k_{res}\,Oc_a}{\tilde{D}_{\mathrm{TGF}-\beta}} \tag{39}$$

where $\alpha_{\mathrm{TGF}-\beta}$ is the concentration of TGF–β in bone matrix and $\tilde{D}_{\mathrm{TGF}-\beta}$ is the TGF–β degradation rate. The concentration of TGF–β is used to define the activator/repressor functions in Eqs. (1)-(4) of the main document. These functions control the upregulation of the differentiation of $\mathrm{Ob_u}$ into $\mathrm{Ob_p}$, the upregulation of osteoclast apoptosis and the downregulation of the differentiation of $\mathrm{Ob_p}$ into $\mathrm{Ob_a}$:

$$\pi^{\mathrm{TGF}-\beta}_{\mathrm{act,Ob_u}} = \pi^{\mathrm{TGF}-\beta}_{\mathrm{act,Oc_p}} = \frac{[\mathrm{TGF}-\beta]}{K^{\mathrm{TGF}-\beta}_{\mathrm{act}} + [\mathrm{TGF}-\beta]} \tag{40}$$

$$\pi^{\mathrm{TGF}-\beta}_{\mathrm{rep,Ob_p}} = \frac{K^{\mathrm{TGF}-\beta}_{\mathrm{rep}}}{K^{\mathrm{TGF}-\beta}_{\mathrm{rep}} + [\mathrm{TGF}-\beta]} \tag{41}$$

with $K^{\mathrm{TGF}-\beta}_{\mathrm{act}}$ and $K^{\mathrm{TGF}-\beta}_{\mathrm{rep}}$ the activation and repression constants, respectively.

7. Proliferation of osteoblast precursors

Let us recall the differential equation of osteoblast precursors.

$$\frac{d\,\mathrm{Ob_p}}{dt} = D_{\mathrm{Ob_u}} \cdot \mathrm{Ob_u} \cdot \pi^{\mathrm{TGF-\beta}}_{\mathrm{act,Ob_u}} - D_{\mathrm{Ob_p}} \cdot \mathrm{Ob_p} \cdot \pi^{\mathrm{TGF-\beta}}_{\mathrm{rep,Ob_p}} + P_{\mathrm{Ob_p}} \cdot \mathrm{Ob_p} \cdot \pi^{\mathrm{Wnt}}_{\mathrm{act,Ob_p}} \tag{42}$$

We can rewrite this equation as:

$$\frac{d\,\mathrm{Ob_p}}{dt} = \mathcal{D}_{\mathrm{Ob_u}} \cdot \mathrm{Ob_u} - \mathcal{D}_{\mathrm{Ob_p}} \cdot \mathrm{Ob_p} + \mathcal{P}_{\mathrm{Ob_p}} \cdot \mathrm{Ob_p} \tag{43}$$

where the terms in the right-hand side correspond, respectively, to the differentiation of $\mathrm{Ob_u}$ into $\mathrm{Ob_p}$, the differentiation of $\mathrm{Ob_p}$ into $\mathrm{Ob_a}$ and the proliferation of $\mathrm{Ob_p}$ at a rate $\mathcal{P}_{\mathrm{Ob_p}}$ which is determined by $P_{\mathrm{Ob_p}}$ and the Wnt–Scl–LRP5/6 signalling pathway through $\pi^{\mathrm{Wnt}}_{\mathrm{act,Ob_p}}$ (see Eq. (42)). As discussed in Buenzli et al. [9], a necessary condition for the $\mathrm{Ob_p}$ population to stay bounded and to converge to a meaningful steady-state (with finite, positive cell densities) is that:

$$\mathcal{P}_{\mathrm{Ob_p}} - \mathcal{D}_{\mathrm{Ob_p}} < 0 \quad \text{as} \;\; t \longrightarrow \infty \tag{44}$$

Following Buenzli et al. [9] $P_{\mathrm{Ob_p}}$ was defined considering a saturation factor:

$$P_{\mathrm{Ob_p}} = \begin{cases} P^0_{\mathrm{Ob_p}} \left(1 - \dfrac{\mathrm{Ob_p}}{\mathrm{Ob_p^{sat}}}\right) & \text{if} \quad \mathrm{Ob_p} < \mathrm{Ob_p^{sat}} \\ 0 & \text{if} \quad \mathrm{Ob_p} \geq \mathrm{Ob_p^{sat}} \end{cases} \tag{45}$$

where $P^0_{\mathrm{Ob_p}}$ is a constant and $\mathrm{Ob_p^{sat}}$ is the maximum concentration of osteoblast precursors above which no proliferation occurs. This saturation and the choice of $P^0_{\mathrm{Ob_p}}$ (see Table 1) ensures that Eq. (44) is fulfilled.

8. Model constants

The model constants, except for those related to the damage and mineralisation algorithms, which were given in the main document, are provided in the following table.

Constant	Value	Units
Cell constants: differentiation, proliferation, apoptosis, activity		
$\mathrm{Ob_u}$	0.01	pM
$\mathrm{Oc_u}$	0.01	pM
$D_{\mathrm{Ob_u}}$	0.083	day^{-1}
$D_{\mathrm{Ob_p}}$	0.185	day^{-1}
$P^0_{\mathrm{Ob_p}}$	2.73	day^{-1}
$\mathrm{Ob_p^{sat}}$	0.005	pM
$D_{\mathrm{Oc_u}}$	0.011	day^{-1}
$D_{\mathrm{Oc_p}}$	0.0196	day^{-1}
$\Delta_{\mathrm{Ob_a}}$	0.212	day^{-1}
$A_{\mathrm{Oc_a}}$	10.0	day^{-1}
η	$4.143 \cdot 10^{-4}$	pM / %[1]
k_{res}	2500	% day^{-1} pM^{-1}
k_{form}	50	% day^{-1} pM^{-1}
RANK-RANKL-OPG signalling pathway		
$\tilde{D}_{\mathrm{OPG}}$	$5.326 \cdot 10^{5}$	day^{-1}
$\tilde{D}_{\mathrm{RANKL}}$	10.132	day^{-1}
$\tilde{D}_{\mathrm{Dmab_{BC}}}$	24.5 *	day^{-1}
$\tilde{D}_{\mathrm{OPG-RANKL}}$	10.132	day^{-1}
$\tilde{D}_{\mathrm{RANK-RANKL}}$	10.132	day^{-1}
$\tilde{D}_{\mathrm{Dmab-RANKL}}$	10.132 *	day^{-1}
$K_{\mathrm{OPG-RANKL}}$	$1.511 \cdot 10^{-2}$	pM
$K_{\mathrm{RANK-RANKL}}$	10	pM
$K_{\mathrm{Dmab-RANKL}}$	10000	pM

Continued on next page

[1]Recall that f_{bm} is expressed in %.

Constant	Value	Units
$N_{\mathrm{Oc_p}}^{\mathrm{RANK}}$	$4.16 \cdot 10^3$	pM RANK / pM cell
$\beta_{\mathrm{OPG,Ob_a}}$	$1.625 \cdot 10^8$	pM OPG / pM cell day^{-1}
$[\mathrm{OPG}]_{\mathrm{max}}$	$1.314 \cdot 10^2$	pM
$[\mathrm{RANKL}]_{\mathrm{max}}$	$3.051 \cdot 10^3$	pM
$\beta_{\mathrm{RANKL,Ot}}$	$5.66 \cdot 10^3$	pM RANKL / pM cell day^{-1}
$\beta_{\mathrm{RANKL,Ob_p}}$	$2.36 \cdot 10^4$	pM RANKL / pM cell day^{-1}
$K_{\mathrm{act,Oc_u}}^{\mathrm{RANKL}}$	16.7	pM
$K_{\mathrm{act,Oc_p}}^{\mathrm{RANKL}}$	3.34	pM
Upregulation of RANKL via damage		
ρ_{dam}	0.04 *	-
α_{dam}	1 *	-
K_{dam}	0.2 *	-
Competitive binding Wnt-Scl-LRP5/6		
$\tilde{D}_{\mathrm{Scl}}^{0}$	1	day^{-1}
$\tilde{D}_{\mathrm{Scl-LRP5/6}}$	50	day^{-1}
$K_{\mathrm{Wnt-LRP5/6}}$	1000	pM
$K_{\mathrm{Scl-LRP5/6}}$	10	pM
$N_{\mathrm{OB_p}}^{\mathrm{LRP5/6}}$	5	pM LRP5/6 / pM cell
$\beta_{\mathrm{Scl,Ot}}$	$2.4 \cdot 10^4$ *	pM Scl / pM cell day^{-1}
[Wnt]	170	pM
$[\mathrm{Scl}]_{\mathrm{max}}$	70	pM
$P_{\mathrm{Scl,d}}$	0	pM day^{-1}
Co-regulation of RANKL via PTH and NO		
λ_s	0.45	-
λ_c	0.9	-
$K_{\mathrm{act}}^{\mathrm{PTH}}$	0.65	pM
$K_{\mathrm{rep}}^{\mathrm{PTH}}$	0.223	pM
$K_{\mathrm{rep}}^{\mathrm{NO}}$	$6.44 \cdot 10^3$	pM
$[\mathrm{NO}]_{\mathrm{max}}$	$2 \cdot 10^8$	pM
β_{PTH}	250	pM day $^{-1}$

Continued on next page

Constant	Value	Units
$\tilde{D}_{\mathrm{PTH}}$	86	day^{-1}
$\beta_{\mathrm{NO,Ot}}$	$3.44 \cdot 10^3$ *	pM NO / pM cell day^{-1}
$\tilde{D}_{\mathrm{NO}}$	$2.1 \cdot 10^{-3}$	day^{-1}
$P_{\mathrm{NO,d}}$	0	pM day^{-1}
	PMO related constants	
$P_{\mathrm{RANKL,max}}$	8 *	pM
γ	0.8 *	-
δ_{PMO}	300 *	days
τ_{PMO}	20	years
	Dmab PK-PD constants *	
k_a	0.17	day^{-1}
k_{el}	$1.15 \cdot 10^{-2}$	day^{-1}
V_c	77.9	ml kg^{-1}
F	1	-
K_m	411	ng ml^{-1}
V_{max}	2672	ng kg^{-1} day^{-1}
ζ	0.85	-
	TGF-β related constants	
$\frac{\alpha_{\mathrm{TGF}-\beta}\, k_{res}}{\tilde{D}_{\mathrm{TGF}-\beta}}$	1	-
$K_{\mathrm{act}}^{\mathrm{TGF}-\beta}$	$5.633 \cdot 10^{-4}$	pM
$K_{\mathrm{rep}}^{\mathrm{TGF}-\beta}$	$1.754 \cdot 10^{-4}$	pM
	Parameters of mechanical regulation	
α_{rep}	1	-
α_{act}	1	-
ρ_{rep}	0	-
ρ_{act}	0	-
δ_{rep}	$9.612 \cdot 10^{-3}$	MPa
δ_{act}	$4.368 \cdot 10^{-3}$	MPa
γ_{rep}	8	-

Continued on next page

Constant	Value	Units
γ_{act}	7	-

Table 1: Values taken for the constants of the PK-PD model. All the constants were taken from the model developed by Martin et al. [1], except those marked with an asterisk.

All the constants in Table 1 were taken from the model developed by Martin et al. [1], except those marked with an asterisk. These changes were motivated by the fact that Martin et al. did not considered damage and the variable mineral content of bone matrix. Hence, those constants needed to be readjusted to reproduce the behaviour of the previous model. So, $\beta_{\mathrm{Scl,Ot}}$ and $\beta_{\mathrm{NO,Ot}}$ were readjusted to achieve the same sclerostin and NO levels. The constants of the block "Upregulation of RANKL via damage" are new since damage were not considered in the previous model. Their values were fitted as in [8], so that the contribution of damage to RANKL production is similar in normal situations (homeostasis) to the production of RANKL by osteoblast precursors (see Eq. (11) in the main document). The constants of the block "Dmab PK-PD constants" are specific of the Dmab PK model and are taken from previous works [8, 10]. The degradation rate of the complex RANKL-Dmab, $\tilde{D}_{\mathrm{Dmab-RANKL}}$, was chosen equal to the degradation rates of the other complex involving RANKL, while ζ and $\tilde{D}_{\mathrm{Dmab_{BC}}}$ were adjusted as explained in section 3.1 of the main document. The constants of the block "PMO related constants" were readjusted as done in [1], i.e. to reproduce the results of the longitudinal experimental study of Nordin et al. [11] on the evolution of the bone mineral density (BMD) in the forearm of post-menopausal women. Martin et al. [1] did not account for variations in mineral content and assumed the bone matrix fraction to evolve in the same way as the BMD. Now that this limitation has been overcome by including the mineral content, it was necessary to readjust the constants.

References

[1] Martin M, Sansalone V, Cooper D, Forwood M, Pivonka P. Mechanobiological osteocyte feedback drives mechanostat regulation of bone in a multiscale computational model. Biomech Model Mechanobiol 2019;18(5):1475–96. doi:10.1007/s10237-019-01158-w.

[2] Pivonka P, Zimak J, Smith DW, Gardiner BS, Dunstan CR, Sims NA, et al. Model structure and control of bone remodeling: A theoretical

study. Bone 2008;43(2):249–63. URL: http://dx.doi.org/10.1016/j.bone.2008.03.025. doi:10.1016/j.bone.2008.03.025.

[3] Pivonka P, Buenzli P, Dunstan C. A systems approach to understanding bone cell interactions in health and disease. In: Gowder SJT, editor. Cell Interaction. IntechOpen; 2012,doi:10.5772/51149.

[4] Ardawi M, Al-Kadi H, Rouzi A, Qari M. Determinants of serum sclerostin in healthy pre- and postmenopausal women. J Bone Miner Res 2011;26(12):2812–22. doi:https://doi.org/10.1002/jbmr.479.

[5] Jastrzebski S, Kalinowski J, Stolina M, Mirza F, Torreggiani E, Kalajzic I, et al. Changes in bone sclerostin levels in mice after ovariectomy vary independently of changes in serum sclerostin levels. J Bone Miner Res 2013;28(3):618–26. doi:https://doi.org/10.1002/jbmr.1773.

[6] Peterson MC, Riggs MM. A physiologically based mathematical model of integrated calcium homeostasis and bone remodeling. Bone 2010;46(1):49–63. doi:10.1016/j.bone.2009.08.053.

[7] Beaupré G, Orr T, Carter D. An approach for time-dependent bone modeling and remodeling–theoretical development. J Orthop Res 1990;8(5):651–61.

[8] Martínez-Reina J, Calvo-Gallego J.L. Pivonka P. Combined effects of exercise and denosumab treatment on local failure in post-menopausal osteoporosis – Insights from bone remodelling simulations accounting for mineralisation and damage. Front Bioeng Biotechnol 2021;9:635056. doi:10.3389/fbioe.2021.635056.

[9] Buenzli P, Pivonka P, Gardiner B, Smith D. Modelling the anabolic response of bone using a cell population model. J Theor Biol 2012;307:42–52. doi:10.1016/j.jtbi.2012.04.019.

[10] Martínez-Reina J, Calvo-Gallego J.L. Pivonka P. Are drug holidays a safe option in treatment of osteoporosis? – Insights from an in silico mechanistic PK–PD model of denosumab treatment of postmenopausal osteoporosis. J Mech Behav Biomed Mater 2021;113:104140. doi:10.1016/j.jmbbm.2020.104140.

[11] Nordin B, Need A, Chatterton B, Horowitz M, Morris H. The relative contributions of age and years since menopause to postmenopausal bone loss. J Clin Endocrinol Metab 1990;70(1):83–8. doi:https://doi.org/10.1210/jcem-70-1-83.
